# FGF21 as a Potential Mediator of Ultra-Processed Food-Associated Metabolic Dysfunction-Associated Steatotic Liver Disease and the Protective Effect of Bilberry Extract

**DOI:** 10.3390/nu18152430

**Published:** 2026-07-25

**Authors:** Yanling Lv, Feiyang Zhao, Yaqi Zhang, Zekun Zheng, Cunpeng Hou, Guanhua Jiang, Shan Lin, Liegang Liu, Liangkai Chen

**Affiliations:** 1 Department of Nutrition and Food Hygiene, Hubei Key Laboratory of Food Nutrition and Safety, School of Public Health, Tongji Medical College, Huazhong University of Science and Technology, Wuhan 430030, China; lvyanling@hust.edu.cn (Y.L.); d202582339@hust.edu.cn (F.Z.); yaqizhang82@163.com (Y.Z.); aaqian990225@gmail.com (Z.Z.); d202582296@hust.edu.cn (C.H.); d202281799@hust.edu.cn (G.J.); d202081512@hust.edu.cn (S.L.); 2 Ministry of Education Key Laboratory of Environment and Health, School of Public Health, Tongji Medical College, Huazhong University of Science and Technology, Wuhan 430030, China

**Keywords:** metabolic dysfunction-associated steatotic liver disease, fibroblast growth factor 21, ultra-processed food, bilberry extract, plasma proteomics

## Abstract

**Background and Purpose:** Ultra-processed food (UPF) intake is a risk factor for metabolic dysfunction-associated steatotic liver disease (MASLD) development, and fibroblast growth factor 21 (FGF21) is a key regulator of hepatic lipid metabolism, but its role in this association remains unclear. We aimed to investigate whether FGF21 mediates the UPF-MASLD relationship in a population-based cohort and whether berry extract (BE) protects against UPF-related liver injury through FGF21 signaling. **Methods:** This study included 29,386 participants with magnetic resonance imaging (MRI)-derived proton density fat fraction (PDFF) and iron-corrected T1 (cT1) from the UK Biobank. UPF intake was classified according to the NOVA system. Log-binomial and generalized linear regression models were used to estimate the associations of UPF with MASLD, PDFF, and cT1, respectively. In vivo, nine-month-old male C57BL/6J mice were fed a baked Western diet (BWD) with bilberry extract (BE, 200 mg/kg/day) or vehicle for 16 weeks. In vitro, the role of FGF21 was examined by knockdown experiments in AML12 hepatocytes. **Results:** UPF consumption was linearly associated with a higher risk of MASLD, with per 10% increment associated with 9% higher risk of MASLD (RR 1.09 [95% CI 1.07–1.10]), as well as dose-dependent increases in PDFF and cT1. Among the 283 plasma proteins associated with PDFF, FGF21 showed the strongest association with UPF intake and accounted for the largest proportion of mediation in the UPF-PDFF association. A significant interaction between UPF and berry intake was observed in MASLD risk (*p* for interaction = 0.018). In the animal model, BE supplementation for 16 weeks alleviated BWD-induced hepatic steatosis, inflammation, and glucose intolerance, while upregulating hepatic FGF21, FGFR1c, and β-Klotho expression and improving mitochondrial function. FGF21 knockdown abrogated BE’s protective effect against lipid accumulation in vitro. **Conclusions:** FGF21 emerged as a potential mediator of the association between UPF consumption and liver fat accumulation. Anthocyanin-rich dietary interventions may offer a promising strategy to prevent MASLD progression.

## 1. Introduction

Metabolic dysfunction-associated steatotic liver disease (MASLD) has emerged as the most prevalent liver disease globally, affecting approximately 38.2% of adults [1]. MASLD encompasses a pathological spectrum from simple steatosis to steatohepatitis, fibrosis, cirrhosis, and hepatocellular carcinoma [2]. Beyond its severe hepatic consequences, MASLD is associated with a range of extrahepatic outcomes, including type 2 diabetes, cardiovascular diseases, and premature death, all of which contribute to the increasing economic burden of the disease [3]. Although the first drug for treating fibrosis in MASLD was approved by the FDA in 2024, the cornerstone of MASLD prevention remains the adoption of a healthy lifestyle, with dietary modifications playing a crucial role [4,5].

With the advancement of social industrialization, the consumption of ultra-processed foods (UPF) has been steadily increasing over the past two decades [6], particularly in high-income countries like the UK, where it now accounts for more than half of total daily energy intake [7,8]. Increasing studies have established the association between UPF consumption and the development of MASLD [9,10,11,12,13]. However, these studies often rely on less precise methods, such as blood biomarkers or hospital records, to define MASLD, and their findings have yet to be corroborated with more sensitive, objective measurements. Moreover, the underlying molecular mechanisms driving these associations also remain unclear.

A key molecular player in the pathogenesis of MASLD is fibroblast growth factor 21 (FGF21), a hepatokine that has garnered attention as a potential therapeutic target for MASLD. Given its short half-life of approximately 0.5–1.5 h [14], long-acting analogs such as pegozafermin and efruxifermin have been developed and have shown significant fibrosis and liver fat regression in multicenter phase 2/3 clinical trials [15,16]. FGF21 is primarily secreted by the liver and elicits its biological functions by binding to its receptor 1c (FGFR1c) and co-receptor β-klotho. These interactions modulate critical processes, including insulin sensitivity, liver lipid homeostasis, and macronutrient metabolism [14]. FGF21 has been implicated as a mediator of the metabolic benefits conferred by healthy dietary patterns [17]. However, its role in mediating UPF-associated hepatic steatosis, and whether modulation of the FGF21 signaling might represent a therapeutic target for mitigating such diet-induced damage, remains poorly understood.

Bilberry (*Vaccinium myrtillus* L.) extract (BE), a natural source of anthocyanin-rich antioxidants, has previously been shown by our group to alleviate oxidative stress and restore metabolic balance [18,19,20]. In diet-induced MASLD models, BE supplementation has been reported to increase the expression of AMPK and PGC-1α, along with reducing markers of steatosis, inflammation, and fibrosis [21,22]. However, the molecular mechanisms underlying BE’s function remain to be further explored. Therefore, we aim to: (1) examine the association between UPF intake and hepatic lipid accumulation, as measured by magnetic resonance imaging (MRI)-proton density fat fraction (PDFF), the most reliable non-invasive biomarker of hepatic steatosis [23]; (2) identify possible molecular pathways linking UPF intake to increased liver fat content, with a particular focus on FGF21 signaling; and (3) explore whether BE can alleviate UPF-related MASLD by modulating the FGF21 signaling pathway and elucidate the underlying mechanisms.

## 2. Materials and Methods

### 2.1. Human Study

#### 2.1.1. Study Population

The UK Biobank study is a large-scale population-based ongoing cohort that began between 2006 and 2010 and enrolled more than 0.5 million adults aged 37 to 73 years old. Detailed information has been described elsewhere [24]. We included participants with at least one 24 h dietary assessment in the UK Biobank. We excluded those with extreme energy intake (<800 or >4200 kcal/day in males and <600 or >3500 kcal/day in females, n = 2228) and those with cardiovascular diseases, cancer, or liver diseases at baseline (n = 36,648). After excluding those without PDFF or liver cT1 data, 29,386 participants were included in the PDFF-related analysis, 18,842 in the liver cT1 analysis, and 17,895 with both PDFF and plasma proteomics data in the proteomics-related analysis (Figure S1).

#### 2.1.2. Exposure Assessment

The 24-h dietary information was collected using the Oxford WebQ between April 2009 and September 2010 at baseline assessment centers, followed by four additional invitations sent via email from February 2011 to February 2012. The web-based questionnaire was validated against interviewer-administered 24 h recall [25] and biomarker studies [26]. The intakes of more than 200 foods and 30 beverages were calculated by multiplying the servings by portion size [27]. For participants with two or more dietary recall data, the average intake was used. We then categorized all foods and beverages into four groups according to the NOVA food classification system [8], i.e., unprocessed or minimally processed foods (NOVA 1), processed culinary ingredients (NOVA 2), processed foods (PF, NOVA 3), and UPF (NOVA 4). The detailed foods and beverages included in each group are shown in Table S1. The weight ratios of UPF to total food and PF to total food were used. We chose the weight ratio rather than the energy ratio to account for UPF that provide little or no energy (e.g., artificial sweetened beverages) or non-nutritional factors related to food processing (e.g., neoformed contaminants and additives) [28]. Berry intake was calculated as the total grams of berries, grapes, tomatoes, and other fruits (e.g., pomegranate, kiwi, and papaya) according to the botanical definition [29].

#### 2.1.3. Outcome Ascertainment

The primary outcome was MRI- detected MASLD, which was operationally defined as the presence of PDFF ≥ 5% [30] along with at least one cardiometabolic risk factor, in accordance with the conceptual framework of EASL-EASD-EASO Clinical Practice Guidelines [4]. The secondary outcomes included MRI-derived PDFF and iron-corrected T1 time (Liver cT1), indicators for liver fat content and fibroinflammatory changes, respectively [31]. The liver MRI scan was performed by LiverMultiScan© (Perspectum Diagnostics Ltd., Oxford, UK) in 2014, and the detailed scan and analysis protocol has been published elsewhere [32]. The PDFF measurement was derived through iterative decomposition of water and fat using echo asymmetry and least-squares estimation, with PDFF calculated as fat/(fat + water) × 100%. For each participant, the average PDFF of nine regions of interest was used.

#### 2.1.4. Plasma Proteomics Profiling

Plasma samples were collected at baseline and stored at −80 °C. Protein profiling was performed on randomly selected samples using Olink technology and the proximity extension assay. Detailed descriptions of sample selection, workflow, quality control, and data processing have been provided elsewhere [33]. We excluded proteins with >20% missing values and included 2911 proteins for analysis. For proteins with <20% missing data, we used the mean value as imputation [33].

#### 2.1.5. Statistical Analysis

The dose–response relationships of UPF and PF with PDFF-diagnosed MASLD were flexibly modeled using the restricted cubic spline (RCS) with three knots (10th, 50th, and 90th). Relative ratio (RR) and 95% confidence interval (CI) of MASLD were estimated using log-binomial regression. The β-coefficients and 95% CI of PDFF and liver cT1 associated with UPF and PF were estimated using the generalized linear regression model. Several covariates were progressively introduced into the models based on a predefined directed acyclic graph (Figure S2). We adjusted for age at the time of the most recent dietary recall (continuous, in years), age at the time of MRI scan (continuous, in years), and sex (male/female) in Model 1. Model 2 included additional adjustments for race (White/non-White), education (college or university, vocational, upper secondary, lower secondary, or others), Townsend deprivation index (TDI, quintiles), smoking status (current, former, never, or unknown), physical activity (0–599, 600–1199, ≥1200 MET-minutes/week, or unknown), alcohol consumption (0, 0.1–10.0, 10.1–20.0, 20.1–30.0, or >30.0 g/day), and energy intake (Kcal/day, quintiles). We did not include body mass index (BMI) in the main analysis as it is more likely a mediator than a confounder. To limit the potential confounding, we included BMI in the model as a sensitivity analysis.

To test the robustness of results, we performed stratified analysis by major confounders, including age (<60/≥60), sex (male/female), TDI (below/above median), current smoker (yes/no), physical activity (<1200/≥1200 MET-minutes/week, according to the WHO physical activity guideline), and daily energy intake (below/above median). We also adjusted for hypertension, dyslipidemia, and diabetes at baseline (as defined in Table S2) to limit confounding by chronic disease. Additionally, we adjusted for baseline liver function (i.e., albumin, gamma-glutamyl transferase, and alanine aminotransferase). To further minimize potential confounding, we applied propensity score matching for age, sex, TDI, education, physical activity, and BMI. To limit the confounding of alcohol intake, we reran the analysis after excluding participants with high alcohol intake (>30 g/day for men and >20 g/day for women).

To assess the modification of berry intake on the association between UPF and MASLD, we tested interactions between berry and UPF intake by including a multiplicative interaction term in the fully adjusted model. The significance of interaction was examined by the Wald test. We further estimated joint associations between UPF and berry intake with MASLD by generating a combined variable with 15 groups (UPF intake quintiles × berry intake tertiles), using the theoretically highest-risk group (i.e., UPF quintile 5, berry tertile 1) as the reference.

For molecular insights into the association between UPF and PDFF, we conducted two analyses using plasma proteomics data to identify key proteins. We first used lasso regression to identify proteins significantly associated with PDFF, a sensitive indicator for liver fat content. Second, we applied generalized linear regression models to establish the associations between UPF intake and those proteins while adjusting for the covariates mentioned above. We then performed mediation analyses to evaluate the role of these proteins in the association between UPF intake and PDFF using the “mediation” package (R software, version 4.3.1), with 1000 simulations for stable estimates [34]. All plasma protein levels included in the model were standardized. In proteomics analyses, all *p*-values were false discovery rate (FDR) adjusted using the Benjamini and Hochberg method. The Kyoto Encyclopedia of Genes and Genomes (KEGG) and Gene Ontology (GO) enrichment analyses were conducted on https://davidbioinformatics.nih.gov, accessed on 2 March 2025.

All statistical analyses were conducted using SAS version 9.4 (SAS Institute Inc., Cary, NC, USA) and R (Version 4.3.1). A two-sided *p* value < 0.05 was considered statistically significant.

### 2.2. Animal Study

#### 2.2.1. Ethics Statement

All animal procedures were conducted according to international guidelines and approved by the Institutional Animal Care and Use Committee at Tongji Medical College, Huazhong University of Science and Technology (ID 4749).

#### 2.2.2. Animal Models

Nine-month-old male C57BL/6J mice were purchased from Vital River Laboratory (Beijing, China) and housed in a specific pathogen-free facility on a 12 h light/dark cycle at a controlled temperature (23 ± 2 °C), with ad libitum access to food and water. After 2 weeks of acclimation, mice were randomly assigned to one of three groups according to body weight (n = 5/group): control, baked Western diet (BWD), and BWD + BE. The BWD was prepared by baking the western diet (XT079B) at 120 °C for 40 min as previously described^35^ and supplemented with 42 g/L fructose. A control diet (XT079B-C) was used for comparison over 16 weeks. BE, provided by BYHEALTH Co., Ltd., was dissolved in sterilized water and administered daily by oral gavage at 200 mg/kg body weight. The control and BWD groups were intragastrically administered sterilized water as the vehicle.

Food was replaced every two days, and daily food intake was calculated. Body weight was recorded weekly. At the end of the intervention, mice were fasted for 14 h and then sacrificed for sample collection. Serum was separated by centrifuging at 3000 rpm for 10 min and stored at −80 °C. Liver, perirenal fat, and brown fat tissues were embedded in paraffin or optimal cutting temperature compound for histological evaluation or snap-frozen in liquid nitrogen and stored at −80 °C for subsequent analyses.

#### 2.2.3. Oral Glucose Tolerance Test (OGTT)

The OGTT was performed as previously described [35]. Following 14 h of fasting, mice were intragastrically administered 1 g/kg body weight glucose (Sigma, St. Louis, MO, USA). Blood glucose was measured at 0, 15, 30, 60, and 120 min post-gavage by a glucometer (Roche, Basel, Switzerland).

#### 2.2.4. Serum Biochemical Measurement and Liver Function Assessment

Serum glucose, total cholesterol (TC), triglyceride, low-density lipoprotein cholesterol (LDL-C), high-density lipoprotein cholesterol (HDL-C), aspartate aminotransferase (ALT), and alanine aminotransferase (AST) were measured using commercially available assay kits according to the manufacturer’s protocols (Elabscience, Wuhan, China).

#### 2.2.5. Enzyme-Linked Immunosorbent Assay (ELISA)

Serum FGF21 and insulin levels were measured using ELISA kits from Elabscience (Cat. No. E-EL-M0029 for FGF21 and E-EL-M3119 for insulin) following the manufacturer’s instructions. Absorbance was measured at 450 nm with a microplate reader (Spark, Tecan, Männedorf, Switzerland).

#### 2.2.6. Quantitative Real-Time Polymerase Chain Reactions (qRT-PCR)

Total RNA was extracted from liver tissue using TRIzol reagent (Invitrogen, Waltham, MA, USA). The concentration of RNA was quantified by a Nanodrop spectrophotometer (Thermo Fisher Scientific, Waltham, MA, USA), and 2 μg of total RNA was reverse-transcribed using RevertAid First Strand cDNA Synthesis Kit (Thermo Fisher Scientific, #K1622). qRT-PCR was performed using PowerUP SYBR Green Master Mix (Thermo Fisher Scientific, A25742) on a QuantStudio™ 7 Flex real-time PCR system (Applied Biosystems, Thermo Fisher Scientific). The relative expression level of mRNA was calculated using the 2^(−ΔΔCt)^ method and normalized to the reference gene (glyceraldehyde-3-phosphate dehydrogenase, GAPDH). Primer sequences applied in RT-qPCR are shown in Table S3.

#### 2.2.7. Histological Examination

Liver tissues were stained with H&E, Oil Red O, F4/80, and Sirius Red according to standard protocols. For quantitative analysis, five randomly selected microscopic fields (upper-left, lower-left, center, upper-right, and lower-right) were captured per section per mouse at 200× magnification. The mean value of these five fields was calculated as the representative measurement for each mouse. Representative images were selected based on their quantified values being closest to the group mean. All quantifications were performed using Image-Pro Plus 6.0 (Rockville, MD, USA) by investigators blinded to group allocation.

#### 2.2.8. Transmission Electron Microscopy (TEM)

Liver tissues were fixed, embedded, and sectioned for transmission electron microscopy according to standard procedures. For mitochondrial morphometric analysis, approximately 15 mitochondria per mouse were randomly selected and measured for morphological parameters (length, width, perimeter, and area) using ImageJ (Bethesda, MD, USA).

#### 2.2.9. Immunoblotting

Proteins were extracted from tissues with lysis buffer (Beyotime, Shanghai, China, P0013) containing phenylmethanesulfonyl fluoride (Beyotime, Shanghai, China, ST506) and phosphatase inhibitor cocktail (Roche, Basel, Switzerland, HY-K0022). The concentration of protein was quantified by a bicinchoninic acid kit (Thermo Fisher Scientific, Waltham, MA, USA, A55864). Proteins were separated on 7.5%−12.5% SDS-PAGE gels and transferred to a 0.2 μm polyvinylidene fluoride membrane (Roche, Basel, Switzerland, 03010040001). The membrane was incubated overnight with specific primary antibodies, followed by a 1 h incubation with horseradish peroxidase-conjugated secondary antibodies. Detailed information on antibodies used in this study is shown in Table S4. Protein bands were visualized by GeneSnap (Syngene, Cambridge, UK) and quantified with ImageJ software.

#### 2.2.10. Cell Culture and Transfection

The mouse AML12 cell line was purchased from the Cell Bank of the Shanghai Institute of Cells, Chinese Academy of Science (Shanghai, China). Cells were maintained in DMEM/F-12 (Gibco, Grand Island, NY, USA, C11330500BT) with Insulin-Transferrin-Selenium media supplement (ThermoFisher, Waltham, MA, USA, 41400045), 40 ng/mL Dexamethasone (Biosharp, Hefei, China, BL171A), and 10% FBS (Gibco, Grand Island, NY, USA, A5669701) in a humidified atmosphere containing 5% CO_2_ at 37 °C. The appropriate concentration and treatment duration of advanced glycation end product–bovine serum albumin (AGE-BSA, Biogradetech, Beijing, China, BGT-CMP-100) were determined using the CCK-8 assay (Dojindo, Kumamoto, Japan, CK04). Oleic acid (Sigma, St. Louis, MO, USA, O1008-5G) was complexed with BSA (Sigma, St. Louis, MO, USA, SRE0098) by incubating for 30 min at 37 °C before being added to the culture media. The shRNA sequence targeting FGF21 was CTCTACACAGATGACGACCAA, and transfection was performed using Lipofectamine 3000 (ThermoFisher, Waltham, MA, USA, L3000001). Transfected cells were then treated with the indicated agent for 24 h.

#### 2.2.11. Statistical Analysis

Data are shown as mean ± standard error of the mean (SEM) or mean ± standard deviation (SD). For comparisons among three groups, one-way analysis of variance (ANOVA) followed by Tukey’s post hoc test was applied for single-time-point measurements (e.g., biochemical parameters, histological scores, and qPCR data). For repeated-measures data (e.g., body weight and OGTT curves), two-way ANOVA was used with group as the between-subjects factor and time as the within-subjects factor, followed by Bonferroni’s post hoc test for multiple comparisons. All in vitro experiments were performed with at least three independent biological replicates, each with three technical replicates. All experiments were repeated at least three times with representative results shown. All data points in the figure represent individual biological replicates. For histological staining quantification and TEM, three out of five mice per group were randomly selected for sectioning and analysis due to limited tissue availability and technical constraints during embedding and sectioning. For immunoblotting and RT-qPCR analysis, four out of five mice per group were randomly selected. The selection was made before quantitative analysis. Statistical analyses were performed using GraphPad Prism 8.0, and a two-tailed *p*-value < 0.05 was considered statistically significant.

## 3. Results

UPF consumption was associated with higher liver fat and inflammation, with FGF21 as a potential mediator and berry intake as a modifying factor.

We included 29,386 participants (mean age 54.5 ± 7.5 years, 52.4% female) and the baseline characteristics across quintiles of UPF consumption are shown in Table 1. Participants with higher UPF consumption were more likely to be male, less educated, less physically active, have a higher BMI, and have higher serum ALT and GGT levels. Baseline characteristics of the analytic sample and the full dietary cohort are shown in Table S5.

**Figure 1:**
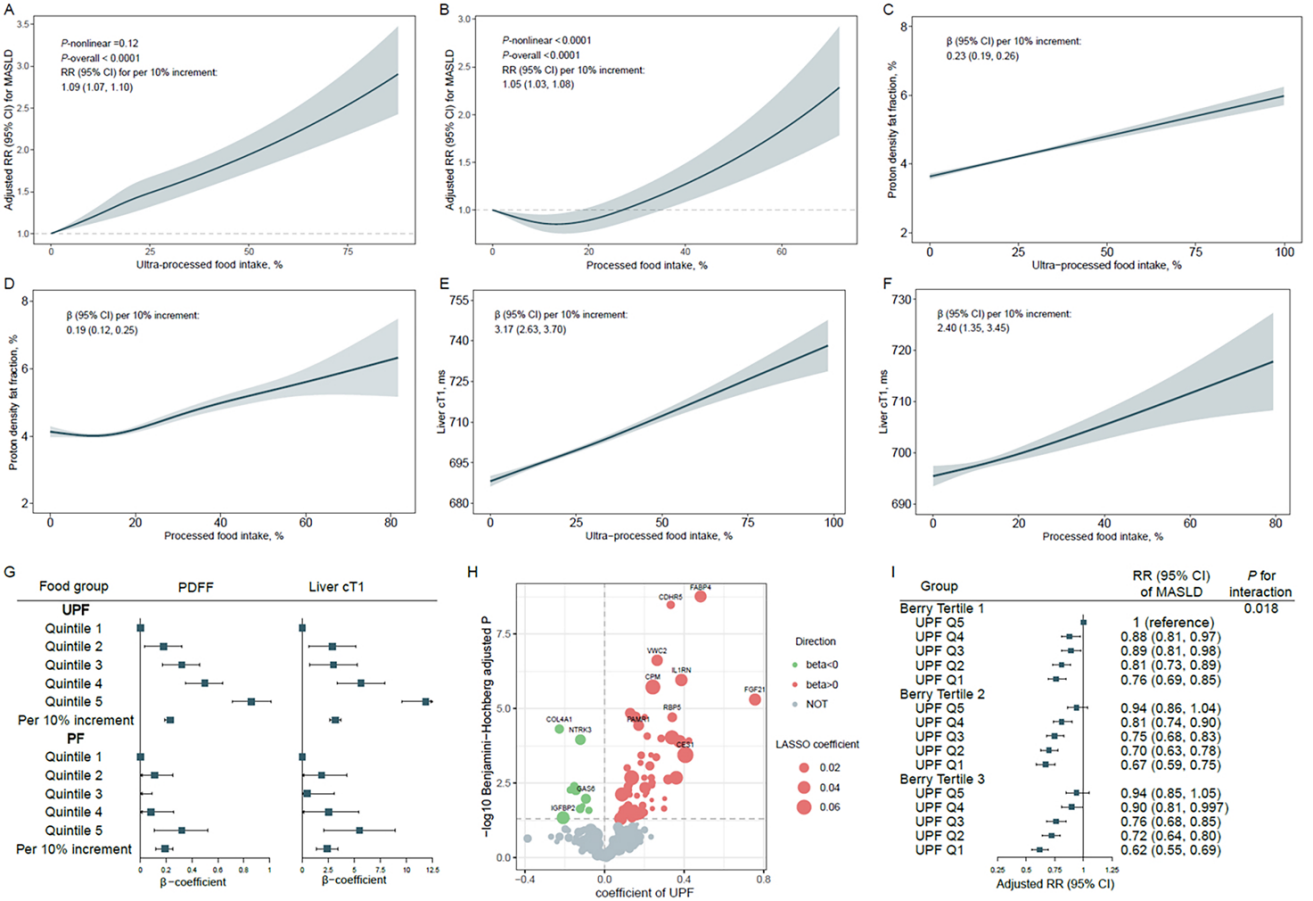
UPF and processed food PF consumption were associated with higher liver fat content and inflammation in general population. (**A**) Restricted cubic spline of UPF intake with the risk of MASLD; (**B**) Restricted cubic spline of PF intake with the risk of MASLD; (**C**) Dose–response relationship between UPF intake and PDFF; (**D**) Dose–response relationship between PF intake and PDFF; (**E**) Dose–response relationship between UPF intake and liver cT1; (**F**) Dose–response relationship between PF intake and liver cT1; (**G**) β-coefficient (95% CI) of UPF and PF intake with PDFF and liver cT1; (**H**) Bubble plot of plasma proteins significantly associated with UPF intake and PDFF; (**I**) Joint association of UPF and berry intake with the risk of MASLD. Abbreviations: CI, confidence interval; MASLD, metabolic dysfunction-associated steatotic liver disease; PDFF, proton density fat fraction; PF, processed food; UPF, ultra-processed food.

After adjusting for demographic and lifestyle confounding factors, both UPF and PF consumption were associated with higher MASLD risk (Figure 1A,B), with multivariable-adjusted RR (95% CI) per 10% increment of 1.09 (1.07–1.10) and 1.05 (1.03–1.08), respectively (Table S6). In addition, both UPF and PF intake were associated with higher PDFF and liver cT1 (Figure 1C–G), and β-coefficients (95% CIs) of PDFF and liver cT1 associated with the per 10% increment of UPF intake were 0.23% (0.19–0.26%) and 3.17 ms (2.63–3.70 ms), respectively (Table S7). Corresponding estimates for PF consumption were 0.19% (95% CI: 0.12–0.25) and 2.40 ms (95% CI: 1.35–3.45) (Table S7). These associations remained consistent across subgroups of age, sex, socioeconomic status, smoking status, physical activity, and energy intake (all *p* for interaction > 0.0014, i.e., 0.05/[3 outcomes × 2 exposures × 6 factors], Tables S8–S10). In sensitivity analyses, these associations remained robust after further adjusting for BMI, liver function, and chronic disorders (Table S11), after excluding participants with excess alcohol consumption (Table S11), as well as in the propensity score-matched subsample (Table S12). Notably, although BMI significantly mediated the association of UPF intake with MASLD risk, PDFF, and liver cT1, the associations of UPF remained statistically significant across all sensitivity analyses (Table S13).

Among 2911 plasma proteins analyzed, 283 were significantly associated with PDFF in LASSO regression (Figure S3). The KEGG and GO enrichment of 283 proteins revealed pathways related to lipid metabolism and inflammation (Figure S4). The top five UPF-associated proteins (*p* < 0.00017, i.e., 0.05/283 proteins) were FGF21, FABP4, GSTA3, CES1, and IL1RN (Figure 1H). Mediation analysis identified FGF21 as the strongest mediator (mediation proportion 39.4%), followed by GSTA3, HNMT, IDUA, and TNFRSF10B (Table S14).

We further examined the association between berry intake and MASLD risk. After adjusting for full covariates and UPF intake, berry intake was significantly associated with lower MASLD risk, as well as lower PDFF and liver cT1 (Tables S15 and S16). In addition, we observed a significant multiplicative interaction between UPF intake and berry consumption on MASLD risk (*p* for interaction = 0.018, Table S17). In joint association, participants in the lowest UPF quintile and highest berry tertile had the lowest MASLD risk (multivariable-adjusted RR = 0.62, 95% CI: 0.55–0.69) compared with those in the highest UPF quintile and lowest berry tertile (Figure 1I).

### 3.1. BE Supplementation Ameliorates BWD Feeding-Induced Liver Damage in Mice

**Figure 2:**
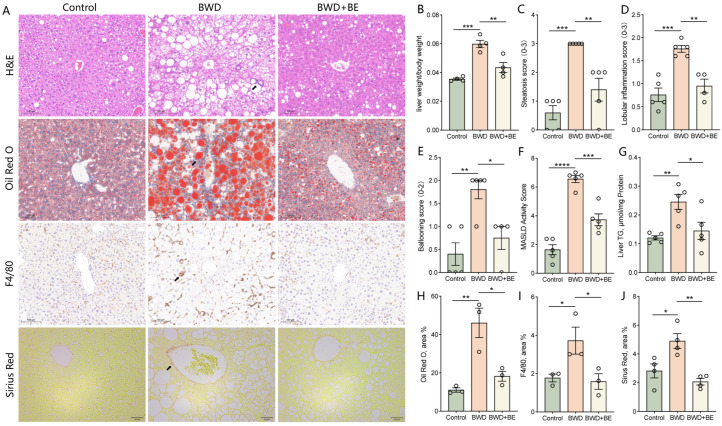
BE supplementation ameliorates BWD feeding-induced liver damage in mice. (**A**) Representative image of H&E, Oil Red O, F4/80, and Sirius Red staining (scale bars, 100 μm); (**B**) Liver weight to body weight ratio; (**C**) Steatosis score based on H&E staining; (**D**) Lobular inflammation score; (**E**) Ballooning score; (**F**) MASLD activity score; (**G**) Liver TG, μmol/mg Protein; (**H**) Oil Red O staining positive area percentage; (**I**) F4/80 staining positive area percentage; (**J**) Sirius Red staining positive area percentage. Data are presented as mean ± SEM, with each data point representing an individual mouse (n = 3–5/group). *p*-value was calculated using one-way ANOVA. *: *p* < 0.05; **: *p* < 0.01; ***: *p* < 0.001; ****: *p* < 0.0001.

**Figure 3:**
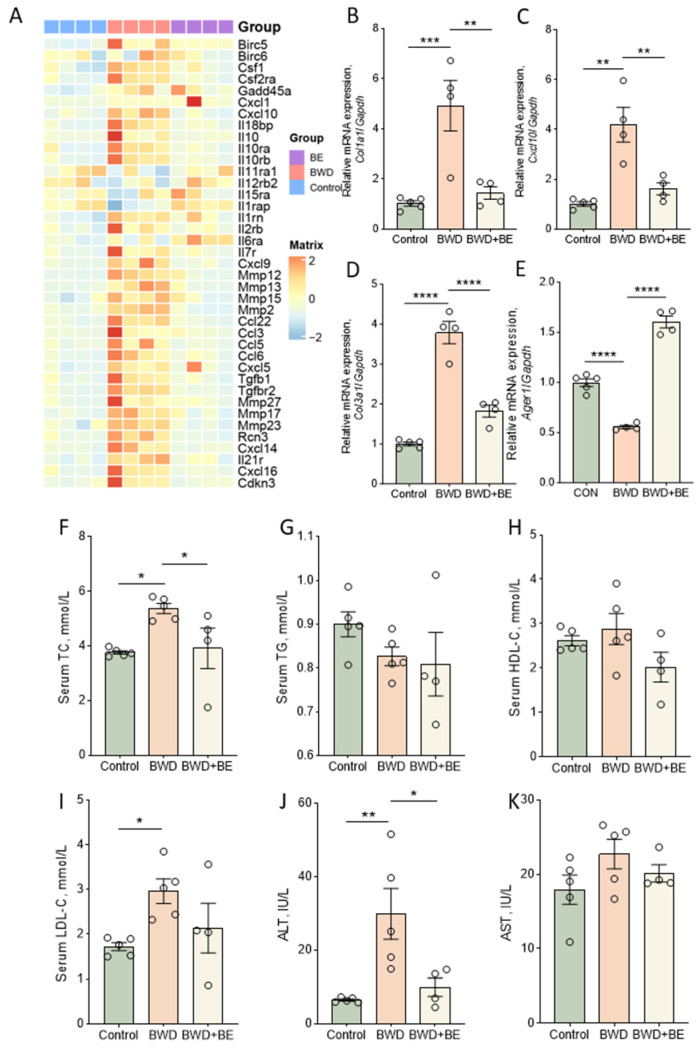
BE supplementation ameliorates BWD feeding-induced liver inflammation in mice. (**A**) Liver transcriptomics; (**B**) Relative mRNA expression of *Col1a1* in liver; (**C**) Relative mRNA expression of *Cxcl10* in liver; (**D**) Relative mRNA expression of *Col3a1* in liver; (**E**) Relative mRNA expression of *Ager1* in liver; (**F**) Serum TC; (**G**) Serum TG; (**H**) Serum HDL-C; (**I**) Serum LDL-C; (**J**) Serum ALT; (**K**) Serum AST. Data are presented as mean ± SEM, with each data point representing an individual mouse (n = 4–5/group). *p*-value was calculated using one-way ANOVA. *: *p* < 0.05; **: *p* < 0.01; ***: *p* < 0.001; ****: *p* < 0.0001.

We then used a mouse model to explore the effect and mechanism of BE supplementation on liver damage. To mimic the baking process frequently used in UPF production, we baked WD at 120 °C for 40 min and supplemented drinking water with 42 g/L fructose. Baseline body weight and food intake were similar among groups (Figure S5A–C). After 16 weeks, BWD feeding led to a more rapid weight gain than the control diet, which was significantly attenuated by BE supplementation (Figure S5D–E). Liver histopathology showed BWD feeding induced marked hepatic steatosis, sinusoidal/perisinusoidal infiltration of inflammatory cells, spotty hepatocyte ballooning, and overall MASLD activity score (Figure 2A–F). The hepatic lipid accumulation and inflammation were further confirmed through the measurement of hepatic triglyceride content and F4/80 expression (Figure 2A,G,H). Sirius red staining showed significant collagen deposition in the BWD group (Figure 2A,J). Notably, BE supplementation markedly reduced steatosis, inflammation, and collagen accumulation caused by BWD (Figure 2A–J). In addition, BE reversed BWD-induced upregulation of inflammation, chemokine, and fibrosis gene expression (Figure 3A–E), and improved serum ALT, TC, and LDL-C levels (Figure 3F–K).

### 3.2. BE Supplementation Improves BWD-Induced Insulin Resistance in Mice

**Figure 4:**
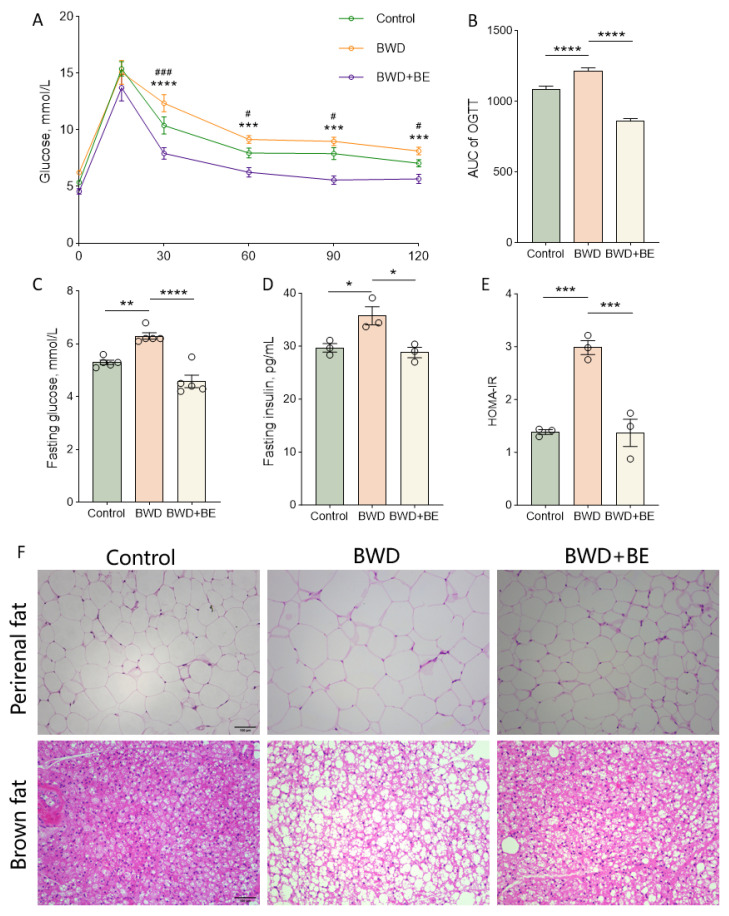
BE supplementation improves BWD-induced insulin resistance in mice. (**A**) Glucose over time after glucose gavage; (**B**) The area under the curve (AUC) of OGTT; (**C**) Fasting serum glucose; (**D**) Fasting serum insulin; (**E**) HOMA-IR index; (**F**) H&E staining of perirenal fat and brown fat (scale bars, 100 μm). Data are presented as mean ± SEM, with each data point representing an individual mouse (n = 3–5/group). *p*-value was calculated using two-way repeated-measures ANOVA for the OGTT time-course data and one-way ANOVA with Tukey’s post hoc test for all other comparisons. *: *p* < 0.05; **: *p* < 0.01; ***: *p* < 0.001; ****: *p* < 0.0001. #: *p* < 0.05; ###: *p* < 0.001 compared glucose in the BWD group with the Control group.

To examine insulin resistance, we performed an OGTT and calculated the HOMA-IR index. Fasting glucose levels were significantly higher in BWD-fed mice but markedly reduced by BE supplementation (Figure 4A,C). Post-load glucose levels and the area under the OGTT curve were elevated in the BWD group, both significantly ameliorated by BE (Figure 4A,B). Similarly, fasting insulin levels and the HOMA-IR index were increased by BWD feeding and normalized following BE supplementation (Figure 4D,E). Histological examination showed that BE mitigated adipocyte hypertrophy in perirenal fat and lipid overaccumulation in brown adipose tissue (Figure 4F).

### 3.3. BE Supplementation Improves FGF21 Signaling in the Liver

**Figure 5:**
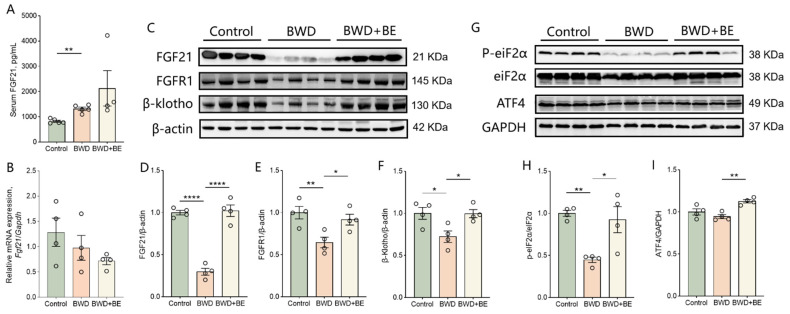
BE supplementation upregulates the hepatic FGF21 signaling pathway. (**A**) Serum FGF21; (**B**) Relative mRNA expression of *Fgf21* in liver; (**C**) Representative blots of FGF21 and its receptors; (**D**) The densitometric analysis of FGF21; (**E**) The densitometric analysis of FGFR1; (**F**) The densitometric analysis of β-Klotho; (**G**) Representative blots of P-eiF2α, eiF2α, and ATF4; (**H**) The densitometric analysis of P-eiF2α/eiF2α; (**I**) The densitometric analysis of ATF4. Data are presented as mean ± SEM, with each data point representing an individual mouse (n = 4–5/group). *p*-value was calculated using one-way ANOVA. *: *p* < 0.05; **: *p* < 0.01; ****: *p* < 0.0001.

Human proteomic analyses above implicated FGF21 as a molecular mediator linking UPF exposure to hepatic steatosis. Accordingly, we evaluated FGF21 and its receptors FGFR1c and β-Klotho in the mouse liver. Serum FGF21 level was significantly increased after 16 weeks of BWD feeding and tended to be higher following the BE supplementation (Figure 5A). Although hepatic FGF21 mRNA expression remained unchanged between groups (Figure 5B), protein levels of FGF21, FGFR1c, and β-Klotho were markedly decreased in the BWD-fed mice and restored by BE supplementation (Figure 5C–F). BE also enhanced hepatic eIF2α phosphorylation and ATF4 expression, the regulators of FGF21 transcription (Figure 5G,H).

To further investigate the effect of FGF21 on the beneficial effect of BE, we knocked down FGF21 expression in AML12 by a shRNA plasmid, and the knockdown efficiency was validated by GFP fluorescence from the plasmid and by immunoblotting (Figure S6A–C). To model UPF-related conditions in vitro, cells were treated with 0.5 mM oleic acid (OA) and 200 μg/mL AGE-BSA, with concentrations optimized based on CCK-8 and triglyceride content assays (Figure S6D,E). The beneficial effect of BE against hepatic lipid accumulation was attenuated following FGF21 knockdown (Figure S6F,G). Taken together, these results suggest that FGF21 may be a key target mediating the protective effect of BE against hepatic lipid accumulation.

### 3.4. BE Supplementation Preserves Mitochondrial Ultrastructure and Function

**Figure 6:**
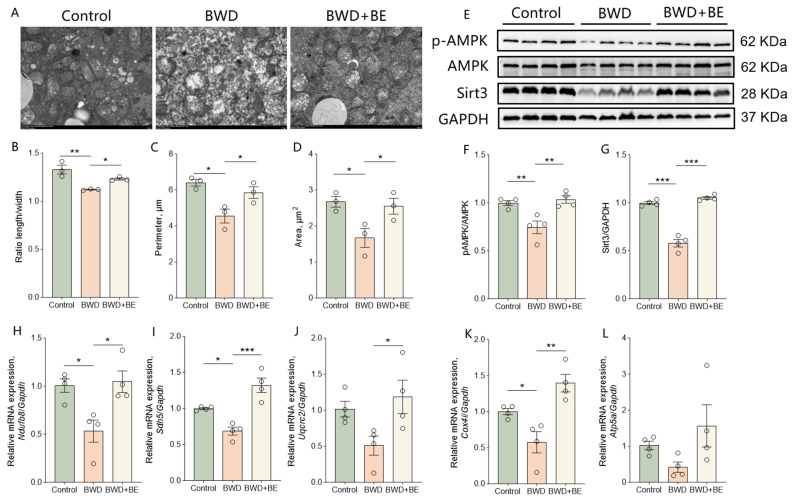
BE supplementation alleviates hepatic mitochondrial ultrastructure and function. (**A**) Representative transmission electron microscope images of mitochondria; (**B**) The ratio of mitochondrial length to width; (**C**) The perimeter of mitochondria; (**D**) The area of mitochondria; (**E**) Representative blots of P-AMPK, AMPK, and Sirt3; (**F**) The densitometric analysis of P-AMPK/AMPK; (**G**) The densitometric analysis of Sirt3; (**H**) Relative mRNA expression of *Ndufb8* in liver; (**I**) Relative mRNA expression of *Sdh5* in liver; (**J**) Relative mRNA expression of *Uqcrc2* in liver; (**K**) Relative mRNA expression of *Cox4i1* in liver; (**L**) Relative mRNA expression of *Atp5a* in liver. Data are presented as mean ± SEM, with each data point representing an individual mouse (n = 3–4 per group). Statistical significance was assessed using one-way ANOVA with Tukey’s post hoc test. *p*-value was calculated using one-way ANOVA. *: *p* < 0.05; **: *p* < 0.01; ***: *p* < 0.001.

Given that mitochondrial function is a potential target for FGF21, we further assessed mitochondrial ultrastructure using transmission electron microscopy. After 16 weeks of feeding, BWD induced mitochondrial cristae rarefaction and swelling, both of which were significantly ameliorated by BE supplementation (Figure 6A). Quantitative analyses confirmed increased mitochondrial length/width ratio, perimeter, and area in BE-treated mice (Figure 6B–D). BE also enhanced AMPK phosphorylation and sirtuin 3 expression (Figure 6E–G). Meanwhile, mRNA expression levels of the five major electron transport chain proteins were significantly restored by BE supplementation (Figure 6H–L).

## 4. Discussion

In this large population-based study, we demonstrated a positive association of UPF intake with MRI-detected liver fat content, inflammation, and MASLD. Human plasma proteomics profiling identified FGF21 as a potential molecular mediator linking UPF consumption to hepatic steatosis. Additionally, the association between UPF and MASLD was modified by berry intake. Complementary experimental studies in mice further confirmed that chronic intake of UPF-like diets induced liver damage, suppressed the hepatic FGF21 pathway, and impaired mitochondrial function. In contrast, these pathologies were substantially ameliorated by BE supplementation.

Our findings reinforce prior epidemiological evidence on the association between UPF consumption and MASLD. In the UK Biobank, Zhao et al. observed a 43% higher risk of MASLD among individuals in the highest quartile of UPF intake after a median follow-up of 8.9 years [12]. However, MASLD diagnosis in this study relied on hospital inpatient records, which largely underestimated the true disease burden [36]. In a Chinese cohort, Zhang et al. reported a 6% higher risk of ultrasound-diagnosed MASLD incidence associated with per SD increment of UPF intake [10]. The association magnitude was slightly lower than our result using PDFF-defined MASLD, which might be explained by the relatively narrow detection range (>2.5% to 20% liver fat content) of ultrasound [37], and this misclassification likely biases results towards null. Using MRI-derived PDFF, the current non-invasive gold standard for quantifying hepatic fat [30], our analysis provided robust evidence for a dose–response relationship between UPF intake and steatosis severity. Notably, we observed a significant interaction between berry intake and UPF consumption on MASLD risk, with the lowest MASLD risk observed in individuals with the lowest UPF consumption and the highest berry intake. Few studies have explored the role of berry intake in modifying the association between UPF consumption and MASLD, while our findings were supported by a previous study reporting that fruit intake modifies UPF and metabolic syndrome [38], as MASLD is the hepatic manifestation of metabolic syndrome.

In mice, BE supplementation markedly improved hepatic steatosis, inflammation, and fibrosis, which aligns with our prior reports highlighting the antioxidant and metabolic benefits of anthocyanin-rich extracts [21,39]. Beyond confirming known effects, our study uncovers additional mechanistic insights into BE. First, our recent study showed that BE effectively reduces diet-induced accumulation of advanced glycation end-products (AGEs) in several organs, including the liver [18]. AGEs are generated abundantly during high-temperature food processing such as baking (the method used to prepare BWD), roasting, or frying [40]. High dietary AGEs intake aggravates MASLD by activating RAGE, thereby triggering the NF-κB signaling cascade and disrupting NRF2 function by neddylation of cullin 3, which in turn suppresses AGEs receptor 1 (AGER1)-mediated AGEs uptake [41]. In our study, BE effectively reversed BWD-induced suppression of hepatic AGER1 expression, suggesting a possible mechanism for BE’s effect on MASLD improvement. However, whether AGER1 directly mediates these benefits requires further validation in vivo. Second, FGF21, a key endocrine regulator of energy homeostasis, exerts its metabolic effects by binding to FGFR1c and β-Klotho. In the present study, BE supplementation not only further elevated circulating FGF21 levels but also restored BWD-suppressed hepatic FGFR1c and β-Klotho expression. Consistent with our findings, the predominant anthocyanin in BE, i.e., cyanidin-3-galactoside [42], has been shown to upregulate FGF21 signaling both in vitro and in vivo [43,44,45]. Nevertheless, given that FGF21 is also a downstream effector of cellular stress responses, the observed elevation in FGF21 signaling after BE treatment may reflect a broader metabolic adaptation rather than a direct pharmacological target of berry anthocyanins. Distinguishing between these possibilities would require time-course experiments and tissue-specific FGF21 manipulation.

Mitochondrial dysfunction is a hallmark of steatohepatitis and a major contributor to oxidative stress and energy dysregulation in MASLD [46]. In our study, BWD feeding caused profound hepatic mitochondrial swelling, mitochondrial cristae disorganization, and lipid accumulation, all of which were reversed by BE supplementation. Under stress conditions, the mitochondrial quality control system, particularly the integrated stress response (ISR), is initiated to repair mitochondrial function [47]. We observed significantly higher eIF2α phosphorylation, ATF4 expression, and FGF21 signaling, which are all components of ISR [47]. In turn, the elevated FGF21 expression maintains mitochondrial function and energy metabolism [14]. In parallel, we observed significantly higher phosphorylation of AMPK and elevated expression of Sirtuin 3, a highly conserved NAD^+^-dependent protein deacetylase that plays a critical role in controlling mitochondrial function [48], indicating elevated mitochondrial function after BE supplementation. These findings raise the possibility that ISR activation contributes to BE’s beneficial effects on mitochondrial homeostasis. However, the ISR serves as an evolutionarily conserved adaptive mechanism that restores cellular homeostasis under stress via eIF2α phosphorylation and ATF4-driven transcriptional reprogramming, but it triggers apoptosis when stress is overwhelming or unresolved [49]. Our data were collected at a single end-point, and thus we cannot distinguish whether the observed ISR activation represents a protective adaptation or an early stress signal that precedes a maladaptive response. Furthermore, the observed increases in AMPK phosphorylation and Sirt3 expression, while consistent with improved mitochondrial function, could also be secondary to changes in global energy status rather than direct effects of BE on these specific pathways. Future time-course studies and loss-of-function experiments are needed to determine whether ISR, AMPK, or Sirt3 are required for BE-mediated mitochondrial protection.

The principal strengths of our study include the integration of high-resolution MRI-based hepatic fat quantification in a large cohort, human plasma proteomics to identify molecular mediators, and in vivo validation in a diet-induced MASLD model. Several limitations need to be clarified. First, although the LASSO model effectively reduced overfitting, its penalization strategy may have excluded other biologically relevant proteins. Second, the 24 h dietary recall may not fully capture long-term dietary intake, and the lack of detailed information on cooking methods or eating-out items could introduce misclassification under the NOVA classification. Nevertheless, the non-differential misclassification would likely attenuate the observed associations towards the null. Third, though cT1 has been validated as a quantitative biomarker for hepatic inflammation and fibrosis and has demonstrated utility in large population-based studies, it is not a standard clinical sequence available on all MRI platforms. Fourth, participants who underwent the MRI scan were those who responded to the invitation and tended to be healthier than non-respondents, which may have introduced selection bias. Fifth, despite adjusting for a wide range of confounders in the regression models according to the DAG, the close clustering of UPF intake with other unfavorable lifestyles and socioeconomic status means that residual and unmeasured confounding cannot be completely ruled out. Sixth, due to the observational nature of this study, the possibility of reverse causation cannot be excluded. Moreover, the mediation analysis is subject to the inherent limitations of observational design, caution is therefore warranted when interpreting the findings as causal. Seventh, the berry intake assessed in the human study reflects habitual dietary exposure to food groups, which is not directly comparable to the bilberry extract intervention used in the animal experiment. Eighth, BE was administered via gavage, and potential interactions with the gut microbiota remain unexplored. Ninth, the small sample size (n = 5 per group) without a prior power calculation may limit our ability to detect modest effect sizes, and may increase the risk of false positives and overestimation of effect magnitudes. Last but not least, though our findings implicate FGF21 as a mediator, its causal role in BE’s protective effects warrants direct validation using genetic or pharmacological modulation.

Several critical questions remain unanswered and warrant further investigation. First, hepatocyte-specific FGF21-deficient mice or pharmacological neutralization using FGF21 antibodies will be essential to determine whether FGF21 is required for BE’s protective effects in vivo. Second, while our in vivo findings with BE are promising, the individual anthocyanin species or metabolites responsible for the observed effects remain unidentified. Identification of the bioactive components and their tissue-specific distribution would facilitate translation toward defined nutritional interventions. Third, the generalizability of our findings to other populations, including different ethnicities, age groups, and individuals with pre-existing liver disease, needs to be established in future prospective cohorts. Fourth, given that BE was administered via gavage, the contribution of gut microbiota to BE’s hepatic effects remains unexplored; future studies using germ-free or microbiota-depleted models could address this gap. Fifth, although we identified FGF21 as a key mediator through plasma proteomics, other proteins identified in the LASSO model may also contribute to the UPF-MASLD association and merit functional validation. Finally, the translational potential of berry-derived anthocyanins as a dietary intervention for MASLD should be evaluated in randomized controlled trials with histological endpoints. Collectively, addressing these questions will help establish whether FGF21-targeted or anthocyanin-based strategies can be translated into clinical practice for MASLD prevention and treatment.

## 5. Conclusions

Our study suggests that UPF consumption is associated with increased hepatic fat content and MASLD risk, with FGF21 identified as a potential mediator linking UPF intake to hepatic steatosis. BE supplementation effectively ameliorated BWD-induced MASLD by restoring mitochondrial and metabolic homeostasis, potentially involving FGF21 signaling. These findings highlight FGF21 as a potential therapeutic target and suggest that anthocyanin-rich nutritional interventions may offer a promising strategy for mitigating the metabolic consequences of UPF consumption, warranting further validation in clinical settings.

## Figures and Tables

**Table 1:** Baseline characteristics of participants by ultra-processed food intake quintiles.

	Ultra-Processed Food Consumption				
Characteristics	Quintile 1 (n = 5877)	Quintile 2 (n = 5877)	Quintile 3 (n = 5878)	Quintile 4 (n = 5877)	Quintile 5 (n = 5877)
Ultra-processed food intake, median (IQR), %	6.8 (4.6, 8.5)	13.0 (11.6, 14.6)	19.4 (17.8, 21.1)	27.3 (25.0, 29.7)	41.2 (36.3, 48.9)
Age, median (IQR), y	58.1 (52.1, 63.2)	58.1 (52.0, 63.7)	57.9 (51.5, 63.6)	57.8 (51.3, 63.3)	56.2 (49.9, 62.6)
Male, n (%)	2674 (45.5)	2735 (46.5)	2794 (47.5)	2816 (47.9)	2964 (50.4)
Townsend Deprivation Index, median (IQR)	−2.4 (−3.8, 0)	−2.6 (−3.9, −0.4)	−2.7 (−4.0, −0.7)	−2.7 (−4.0, −0.8)	−2.6 (−3.9, −0.6)
Deprivation fifth, n (%)					
First (least deprived)	1086 (18.5)	1133 (19.3)	1226 (20.9)	1226 (20.9)	1182 (20.1)
Second to fourth	3390 (57.6)	3546 (60.3)	3586 (61.0)	3565 (60.7)	3557 (60.5)
Fifth (most deprived)	1398 (23.8)	1198 (20.4)	1061 (18.1)	1077 (18.3)	1133 (19.3)
Unknown	3 (0.1)	0 (0)	5 (0.1)	9 (0.2)	5 (0.1)
Education					
College or university	3424 (58.3)	3293 (56.0)	3025 (51.5)	2778 (47.3)	2524 (43.0)
Vocational	470 (8.0)	523 (8.9)	534 (9.1)	569 (9.7)	626 (10.7)
Upper secondary	732 (12.5)	762 (13.0)	817 (13.9)	818 (13.9)	855 (14.6)
Lower secondary	1036 (17.6)	1056 (18.0)	1258 (21.4)	1401 (23.8)	1559 (26.5)
Others	200 (3.4)	233 (4.0)	233 (4.0)	296 (5.0)	302 (5.1)
Unknown	15 (0.3)	10 (0.2)	11 (0.2)	15 (0.3)	11 (0.2)
Alcohol consumption, median (IQR), g/day	17.1 (0, 36.2)	13.4 (0, 29.7)	12.2 (0, 27.1)	9.4 (0, 24.7)	4.7 (0, 18.1)
Current smoker, n (%)	325 (5.5)	288 (4.9)	311 (5.3)	316 (5.4)	419 (7.1)
Body mass index, median (IQR), kg/m^2^	25.1 (22.9, 27.8)	25.4 (23.2, 28.1)	25.7 (23.4, 28.5)	26.1 (23.7, 28.8)	26.6 (24.1, 29.6)
Total physical activity, MET-mins/week					
0–599	778 (13.2)	808 (13.8)	875 (14.9)	896 (15.3)	1112 (18.9)
600–1199	901 (15.3)	982 (16.7)	948 (16.1)	949 (16.2)	961 (16.4)
≥1200	3437 (58.5)	3316 (56.4)	3231 (55.0)	3155 (53.7)	2884 (49.1)
Unknown	761 (13.0)	771 (13.1)	824 (14.0)	877 (14.9)	920 (15.7)
Energy intake, median (IQR), kcal/d	1924.4 (1619.5, 2259.9)	2027.5 (1732.1, 2382.7)	2076.8 (1771.2, 2419.9)	2067.0 (1759.8, 2433.3)	2042.5 (1711.9, 2409.3)
Alanine aminotransferase, median (IQR), U/L	19.1 (14.7, 25.4)	19.2 (14.9, 25.7)	19.3 (14.8, 26.3)	19.6 (15, 26.5)	20.2 (15.2, 27.9)
Albumin, median (IQR), g/L	45.6 (43.9, 47.3)	45.4 (43.8, 47.2)	45.4 (43.8, 47.1)	45.3 (43.7, 47)	45.4 (43.7, 47.1)
Gamma glutamyltransferase, median (IQR), U/L	23.2 (16.9, 34.8)	23.3 (16.9, 35.4)	24.0 (17.2, 36.4)	24.1 (17.6, 36.5)	24.9 (18.0, 37.3)
Dyslipidemia, n (%)	2292 (39.0)	2445 (41.6)	2561 (43.6)	2644 (45.0)	2841 (48.3)
Hypertension, n (%)	2581 (43.9)	2576 (43.8)	2692 (45.8)	2676 (45.5)	2692 (45.8)
Diabetes, n (%)	160 (2.7)	165 (2.8)	171 (2.9)	217 (3.7)	250 (4.3)

Abbreviations: IQR, interquartile range; MET, metabolic equivalent of task; SD, standard deviation; UPF, ultra-processed food.

## Data Availability

Data from the UK Biobank are publicly available to all researchers upon application in accordance with the UK Biobank data access protocol (https://ukbiobank.ac.uk/enable-your-research/register, accessed on 29 June 2025). This research has been conducted using the UK Biobank Resource under Application 63454.

## Supplementary Materials

The following supporting information can be downloaded at: https://www.mdpi.com/article/10.3390/nu18152430/s1, Figure S1. The flow chart of the present study; Figure S2. Priori defined directed acyclic graph; Figure S3. The LASSO coefficient path and LASSO regularization path; Figure S4. KEGG and GO enrichment of 283 proteins; Figure S5. The body weight and food intake of mice during 16 weeks of feeding and BE intervention; Figure S6. BE reduces hepatic lipid accumulation through FGF21. Table S1. Classification of food items in the UK Biobank according to NOVA Food Classification System; Table S2. Definitions of chronic diseases in the UK Biobank; Table S3. Primer sequence of RT-qPCR; Table S4. Detailed information on antibodies; Table S5. Baseline characteristics of participants with and without PDFF data; Table S6. Associations of UPF and PF intake with MASLD risk; Table S7. β-coefficients (95%CI) of PDFF and liver cT1 associated with UPF and PF intake; Table S8. Stratified analyses by major confounders of association between UPF and PF intake and MASLD risk; Table S9. Stratified analyses by major confounders of association between UPF and PDFF and liver cT1; Table S10. Stratified analyses by major confounders of association between PF and PDFF and liver cT1; Table S11. Sensitivity analysis of further adjusting for BMI, baseline chronic diseases, and baseline liver function in the model; Table S12. Sensitivity analysis of Propensity Score Matching; Table S13. The mediation of BMI in the association of UPF with MASLD risk, PDFF and liver cT1; Table S14. The association of 283 plasma proteins with UPF intake and the mediation in UPF-PDFF association; Table S15. Relative ratio (95% CI) of MASLD risk associated with berry intake; Table S16. β-coefficients (95%CI) of PDFF and liver cT1 associated with berry intake; Table S17. Stratified analyses by berry intake of association between UPF and PF intake and MASLD risk.

## Abbreviations

AGER1, advanced glycation end-products receptor 1; AGEs, advanced glycation end-products; ALT, aspartate aminotransferase; AST, alanine aminotransferase; BE, Bilberry extract; BMI, body mass index; BWD, baked Western diet; CI, confidence interval; ELISA, Enzyme-linked immunosorbent assay; FDR, false discovery rate; FGF21, Fibroblast growth factor 21; FGFR1c, Fibroblast growth factor Receptor 1c; GO, Gene Ontology; HDL-C, high-density lipoprotein cholesterol; ISR, integrated stress response; KEGG, Kyoto Encyclopedia of Genes and Genomes; LDL-C, low-density lipoprotein cholesterol; MASLD, metabolic dysfunction-associated steatotic liver disease; MRI, magnetic resonance imaging; OA, oleic acid; OGTT, Oral glucose tolerance test; PDFF, proton density fat fraction; PF, processed foods; qRT-PCR, Quantitative real-time polymerase chain reactions; RCS, restricted cubic spline; RR, Relative ratio; RR, Relative ratio; SD, standard deviation; SEM, standard error of the mean; TC, total cholesterol; TDI, Townsend deprivation index; UPF, Ultra-processed food.
