# Supplementary material for: FGF21 as a Potential Mediator of Ultra-Processed Food-Associated Metabolic Dysfunction-Associated Steatotic Liver Disease and the Protective Effect of Bilberry Extract
Source: Nutrients. 2026 Jul 25;18(15):2430. doi: 10.3390/nu18152430 (PMC13468626; doi:10.3390/nu18152430)
Supplement: Supplementary file 1 [file nutrients-18-02430-s001.zip › nutrients-4380249-supplementary.pdf]

Supplementary materials

Figure S1. The flow chart of the present study.
Figure S2. Priori defined directed acyclic graph
Figure S3. The LASSO coefficient path and LASSO regularization path
Figure S4. KEGG and GO enrichment of 283 proteins
Figure S5. The body weight and food intake of mice during 16 weeks of feeding and BE intervention.
Figure S6. BE reduces hepatic lipid accumulation through FGF21.

Table S1. Classification of food items in the UK Biobank according to NOVA Food Classification System
Table S2. Definitions of chronic diseases in the UK Biobank
Table S3. Primer sequence of RT-qPCR
Table S4. Detailed information on antibodies
Table S5. Baseline characteristics of participants with and without PDFF data.
Table S6. Associations of UPF and PF intake with MASLD risk
Table S7. β-coefficients (95%CI) of PDFF and liver cT1 associated with UPF and PF intake
Table S8. Stratified analyses by major confounders of association between UPF and PF intake and MASLD risk
Table S9. Stratified analyses by major confounders of association between UPF and PDFF and liver cT1
Table S10. Stratified analyses by major confounders of association between PF and PDFF and liver cT1
Table S11. Sensitivity analysis of further adjusting for BMI, baseline chronic diseases, and baseline liver function in the model
Table S12. Sensitivity analysis of Propensity Score Matching
Table S13. The mediation of BMI in the association of UPF with MASLD risk, PDFF and liver cT1
Table S14. The association of 283 plasma proteins with UPF intake and the mediation in UPF-PDFF association
Table S15. Relative ratio (95% CI) of MASLD risk associated with berry intake
Table S16. β-coefficients (95%CI) of PDFF and liver cT1 associated with berry intake
Table S17. Stratified analyses by berry intake of association between UPF and PF intake and MASLD risk

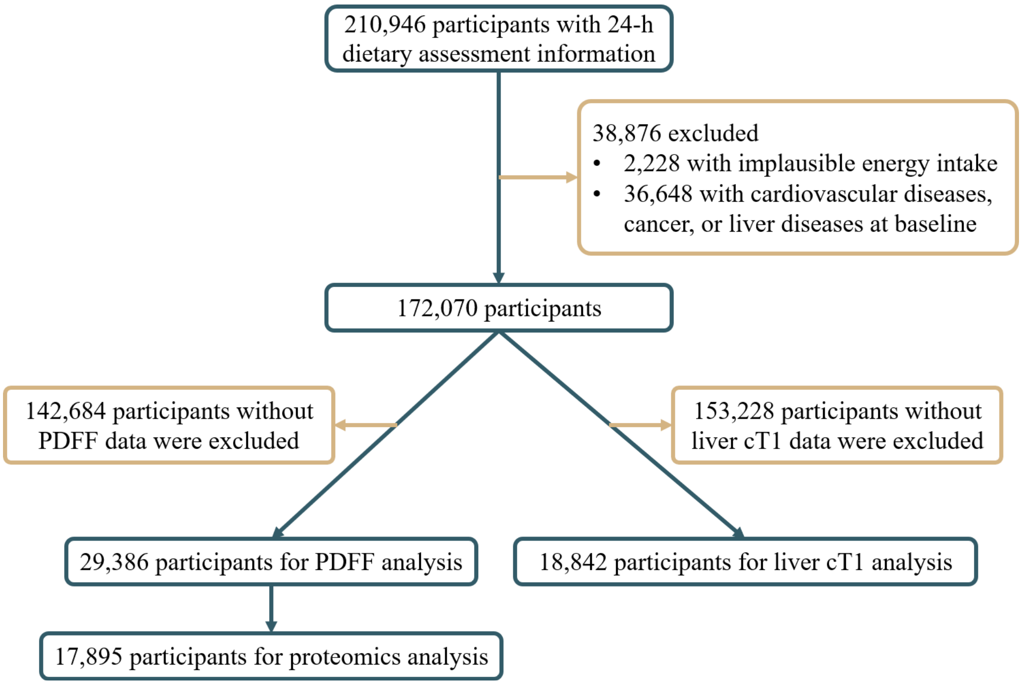

Figure S1. The flow chart of the present study

Abbreviation: PDFF, proton density fat fraction

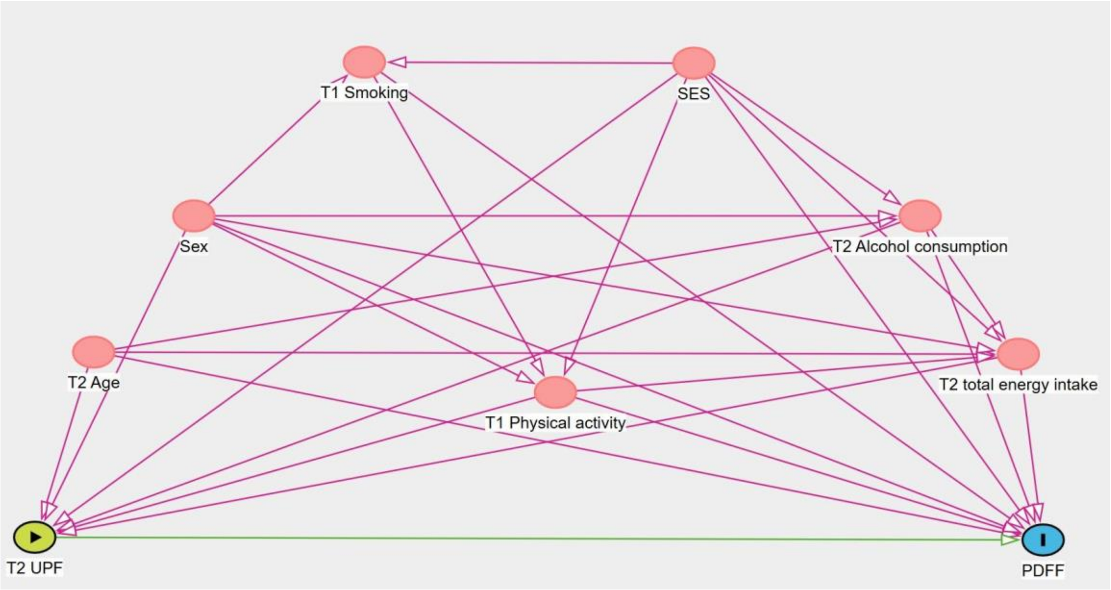

Figure S2. Priori defined directed acyclic graph

T1, covariates collected in enrollment (2006-2010); T2, covariates collected in dietary assessment. SES included Townsend deprivation index and education level.

Abbreviations: PDFF, proton density fat fraction; SES; socioeconomic status; UPF, ultra-processed food.

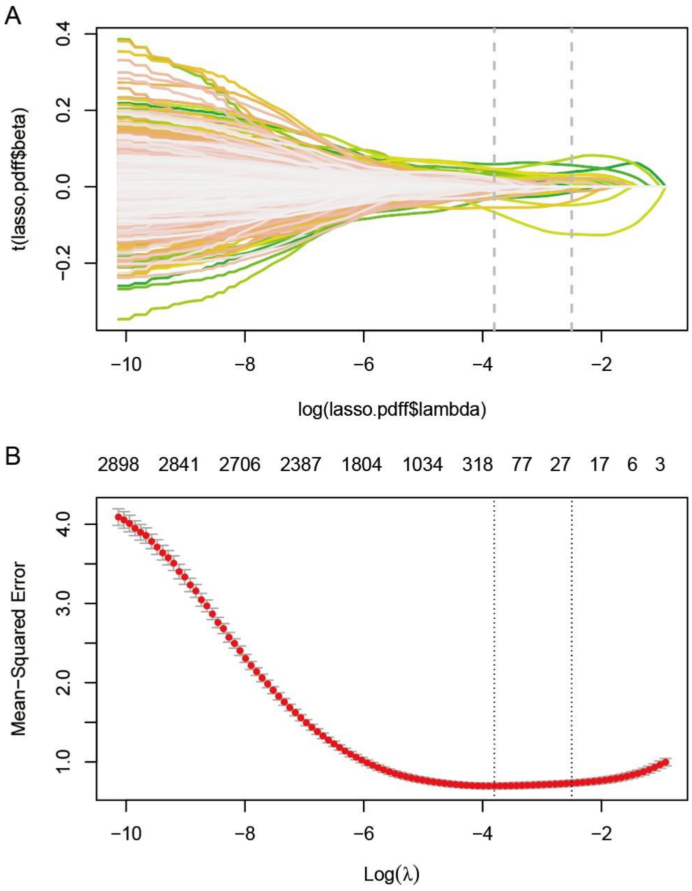

Figure S3. The LASSO coefficient path and LASSO regularization path

A: LASSO coefficient path

B: LASSO regularization path

Abbreviation: LASSO, Least Absolute Shrinkage and Selection Operator

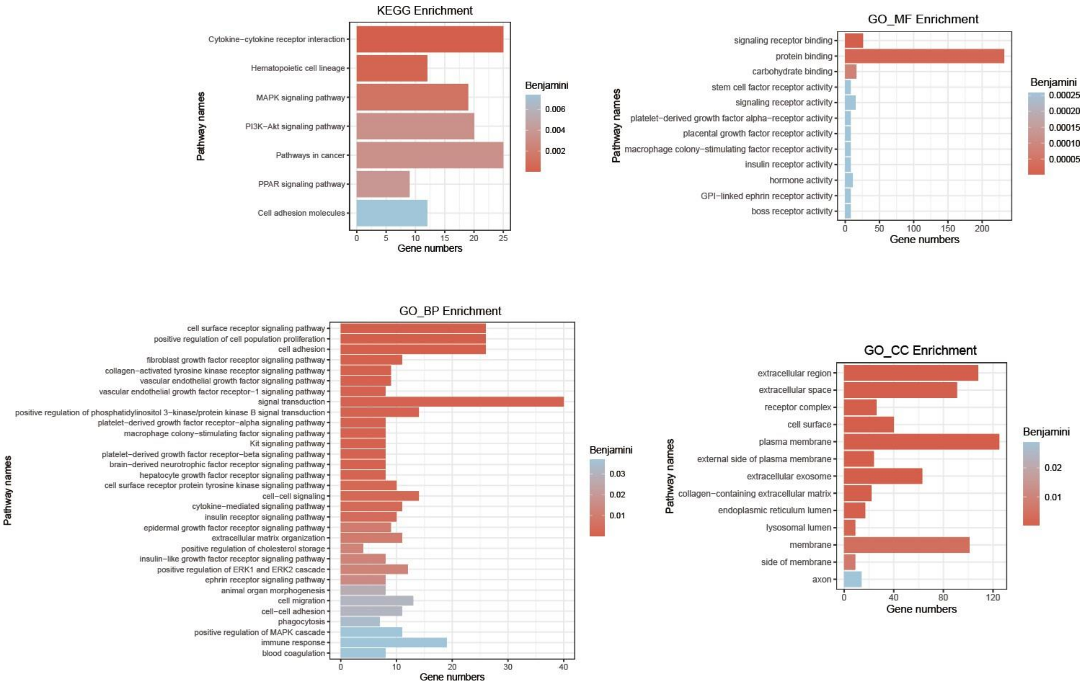

Figure S4. KEGG and GO enrichment of 283 proteins

The significant KEGG and GO enrichment pathways of LASSO regression identified 283 plasma proteins. The significance thresholds for the two-sided *P* values were FDR adjusted using the Benjamini-Hochberg method.

Abbreviations: FDR, false discovery rate; GO, Gene Ontology; KEGG, Kyoto Encyclopedia of Genes and Genomes; LASSO, least absolute shrinkage and selection operator.

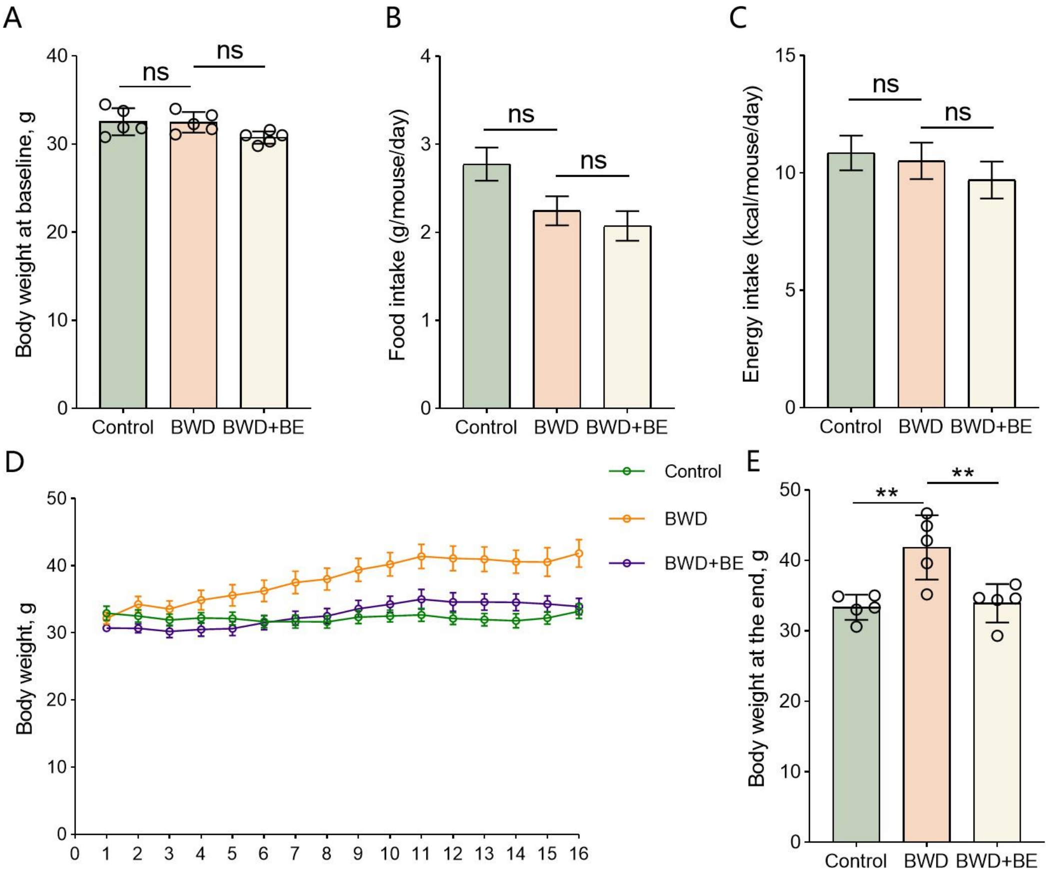

Figure S5. The body weight and food intake of mice during 16 weeks of feeding and BE intervention.

A: Change of body weight during feeding; B: Body weight at baseline; C: Average food intake; D: Average energy intake; E: Body weight at the end of feeding

Data were shown as mean ± SD; ***P*<0.01; ns: not significant

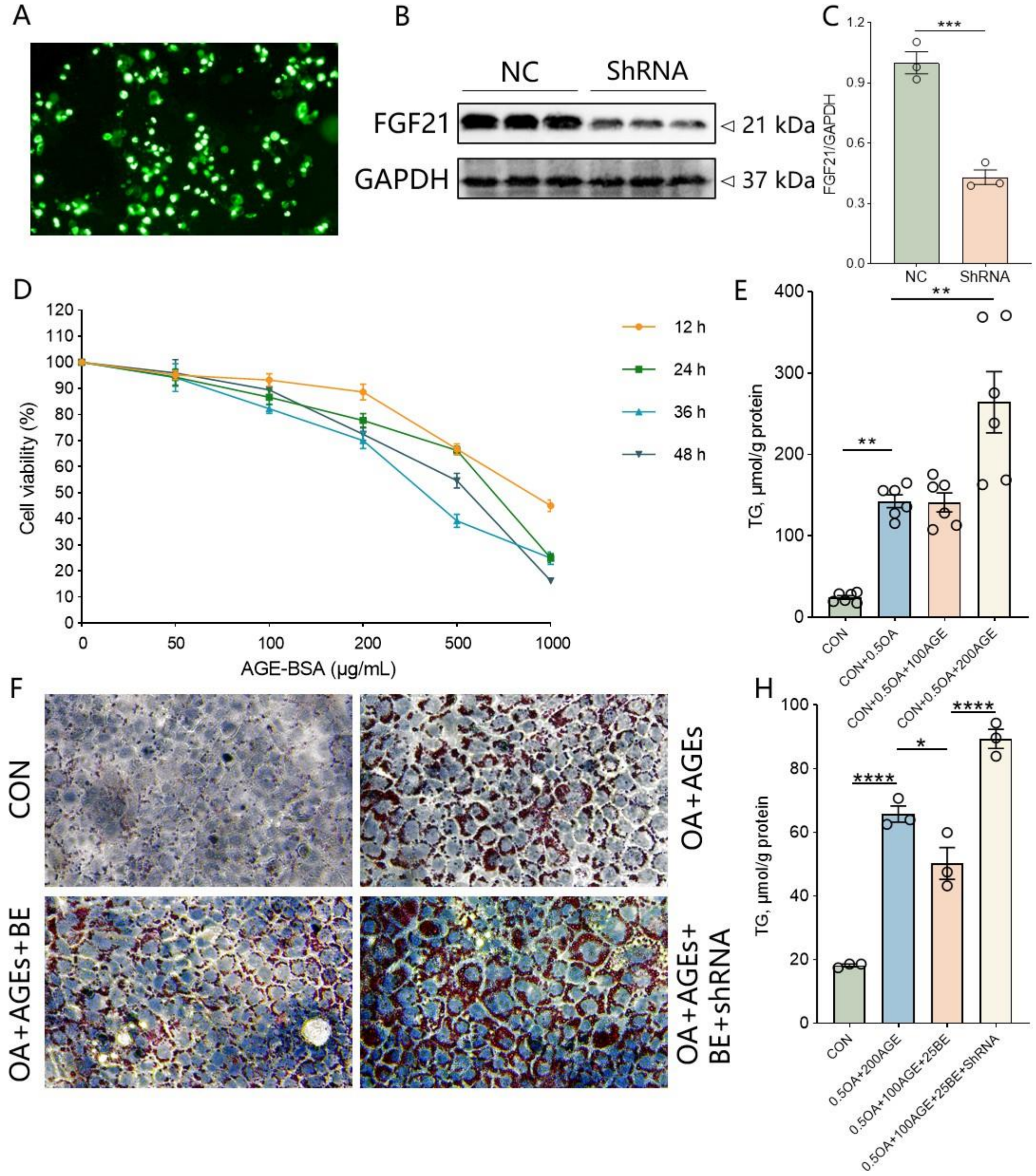

Figure S6. BE reduces hepatic lipid accumulation through FGF21.

A: Rpresentative image of immunofluorescence of GFP; B&C, Rpresentative blots of FGF21 in AML12 cells and densitometric analysis; D, Cell viability of AML12 of different AGE-BSA concentration and treatment duration; E, Triglycerid content of AML12; F, Oil Red O staining of AML12; H, Triglycerid content of AML12. Data were shown as mean ± SD; *P<0.05; **P<0.01; ***P<0.001; ****P<0.0001.

Table S1. Classification of food items in the UK Biobank according to NOVA Food Classification System

Classification	Food items
Unprocessed or minimally processed foods (NOVA 1)	orange juice, grapefruit juice, pure fruit/vegetable juice, filtered coffee, cappuccino, latte, espresso, other coffee, standard tea, rooibos tea, green tea, herbal tea, other tea, milk, porridge, plain cereal, bran cereal, whole-wheat cereal, other cereal, whole egg, other egg, white pasta, wholemeal pasta, white rice, brown rice, couscous, other grain, unsalted peanuts, unsalted nuts, seeds, olives, beef, pork, lamb, poultry, liver, other meat, oily fish, white fish, prawns, lobster/crab, shellfish, other fish, tofu, pulses, boiled/baked potatoes, mixed vegetable, vegetable pieces, coleslaw, side salad, avocado, broad bean, green bean, beetroot, broccoli, butternut squash, cabbage/kale, carrot, cauliflower, celery, courgette, cucumber, garlic, leek, lettuce, mushroom, onion, parsnip, pea, sweet pepper, spinach, sprouts, sweetcorn, sweet potato, fresh tomato, tinned tomato, turnip/swede, watercress, other vegetables, prune, dried fruit, mixed fruit, apple, banana, berry, cherry, grapefruit, grape, mango, melon, orange, satsuma, peach/nectarine, pear, pineapple, plum, and other fruit
Processed Culinary Ingredients (NOVA 2)	sugar, butter/margarine
Processed foods (NOVA 3)	red wine, rose wine, white wine, beer/cider, low fat hard cheese, hard cheese, soft cheese, blue cheese, low fat cheese spread, cheese spread, cottage cheese, feta, mozzarella, goat's cheese, other cheese, omelette, scotch egg, sliced bread, baguette, naan bread, garlic bread, sushi, salted peanuts, salted nuts, homemade soup, bacon, ham, tinned tuna, mashed potato, and stewed fruit
Ultra-processed foods (NOVA 4)	low calorie drink, fizzy drink, squash, fruit smoothie, dairy smoothie, instant coffee, artificial sweetener, flavoured milk, low calorie hot chocolate, hot chocolate, other drink, fortified wine, spirits, other alcohol, muesli, oat crunch, sweetened cereal, intake of artificial sweetener added to cereal, eggs in sandwiches, bap, bread roll, crispbread, oatcakes, other bread, snackpot, double crust pastry, single crust pastry, crumble, pizza, pancake, scotch pancake, Yorkshire pudding, Indian snacks, croissant, Danish pastry, scone, yogurt, ice-cream, milk-based pudding, other milk-based pudding, soya dessert, fruitcake, cake, doughnut, sponge pudding, cheesecake, other dessert,

	chocolate bar, white chocolate, milk chocolate, dark chocolate, chocolate-covered raisin, chocolate sweet, diet sweets, sweets, chocolate-covered biscuits, chocolate biscuits, sweet biscuits, cereal bar, other sweets, crisp, savoury biscuits, cheesy biscuits, other savoury snack, powdered/instant soup, canned soup, sausage, crumbed or deep-fried poultry, breaded fish, battered fish, vegetarian sausages/burgers, Quorn, other vegetarian alternative, baked bean, and fried potatoes

Table S2. Definitions of chronic diseases in the UK Biobank

	ICD-9	ICD-10	OPCS-4	Self-reported (Field ID)	Examination
Dyslipidemia	272	E78		6153, 6177, 20002, 20003	30760, 30870
Hypertension	401, 402, 403, 404, 405	I10, I11, I12, I13, I15, O10, O11		6150, 2966, 6153, 6177, 20002	4079, 4080, 93, 94
Diabetes Mellitus	250	E10-E14		2443, 2976, 6153, 6177, 20002	30740, 30750
Cardiovascular disease*	410, 411, 412, 413, 414, 428, 4280, 4281, 4289, 430, 431, 434, 4340, 4341, 4349, 436	I20-25, I50, I500, I501, I509, I60, I61, I63, I64	A052-A054, K40-K46, K49, K50, K75, L351, L353, L343	3894, 6150, 6153, 6177, 20004, 20002	6150, 2966, 6153, 6177, 20002
Cancer		C00-C97, D00-D48, O00-O08		2453, 20001	

*Cardiovascular disease is composed of coronary heart disease, stroke, and heart failure.

Abbreviations: ICD, International Classification of Diseases; OPCS-4, Office of Population Censuses and Surveys Classification of Interventions and Procedures, version 4.

Table S3. Primer sequence of RT-qPCR

Gene		Sequence（5' to 3'）
Col1a1	Forward	AGCACGTCTGGTTTGGAGAG
	Reverse	GACATTAGGCGCAGGAAGGT
Col3a1	Forward	GTCTGGTGGCTTTTCACCCT
	Reverse	AGTTCGGGGTGGCAGAATTT
Cxcl10	Forward	CCAAGTGCTGCCGTCATTTT
	Reverse	AGCTTCCCTATGGCCCTCAT
Ndufb8	Forward	CTTGTCCTTCCCTCTACGTGC
	Reverse	TCCCCAGTGAGATGACCTATGA
Sdh5	Forward	CTGGCCGGTATGTGAGTGTT
	Reverse	AACTGATGCTGATTCCCGCT
Uqcrc2	Forward	AACTGCTAGAGCCATGAAGC
	Reverse	GTTTTAACCTTCGGGGCAACTT
Cox4i1	Forward	CTTCCCTGATTCCCGCGAT
	Reverse	GACCGCCCTCTACACCCTG
Atp5a	Forward	CAGGAGAGGGGCGGTACTT
	Reverse	GACGACTGGCACAAAATGGC
Fgf21	Forward	TACCCCATCCCTGACTCCAG
	Reverse	ACCACTGTTCCATCCTCCCT
Ager1	Forward	TCCCTACTATGCCAGTGCCT
	Reverse	CCGACCGATCCATTCGTGAT
Gapdh	Forward	CATGGCCTTCCGTGTTCCTA
	Reverse	CCTGCTTCACCACCTTCTTGAT

Table S4. Detailed information on antibodies

Antibody	Dilution	Source	Company
GAPDH	1:10000	Rabbit	CST#2118
β-actin	1:10000	Mouse	Proteintech No. 66009-1-Ig
SIRT3	1:1000	Rabbit	CST#5490
FGF21	1:1000	Rabbit	Abcam#ab171941
β-klotho	1:2000	Goat	R&D#AF2619
FGFR1	1:1000	Rabbit	CST#9740
AMPK	1:1000	Rabbit	CST#5831
P-AMPK (Thr172)	1:1000	Rabbit	CST#2535
eiF2α	1:1000	Rabbit	CST#5324
P-eiF2α (Ser51)	1:1000	Rabbit	CST#3597
ATF4	1:1000	Mouse	Proteintech No. 60035-1-Ig
HRP-conjugated Goat Anti-Rabbit IgG	1:10000	Goat	Proteintech No. SA00001-2
HRP-conjugated Goat Anti-Mouse IgG	1:10000	Goat	Proteintech No. SA00001-1
HRP-conjugated Rabbit Anti-Goat IgG	1:10000	Rabbit	Proteintech No. SA00001-4

Table S5. Baseline characteristics of participants with and without PDFF data.

Characteristics	Participants with dietary information (n=208,718)	Participants with dietary information and PDFF (n=29,386)
Ultra-processed food intake, mean (SD), %	19.8 (11.6, 30.6)	19.4 (11.6, 29.7)
Age, mean (SD), y	59.7 (52.1, 65.0)	57.7 (51.3, 63.3)
Male, n (%)	93,640 (44.9)	13983 (67.6)
Townsend Deprivation Index, median (IQR)	-2.3 (-3.7, 0.1)	-2.6 (-3.9, -0.5)
Deprivation fifth, n (%)		
First (least deprived)	41,635 (20.0)	5,853 (19.9)
Second to fourth	125,108 (59.9)	17,644 (60.0)
Fifth (most deprived)	41,715 (20.0)	5,867 (20.0)
Unknown	260 (0.1)	22 (0.1)
Education		
College or university	88,979 (42.6)	15,044 (51.2)
Vocational	21,753 (10.4)	2,722 (9.3)
Upper secondary	27,234 (13.1)	3,984 (13.6)
Lower secondary	51,956 (24.9)	6,310 (21.5)
Others	17,783 (8.5)	1,264 (4.3)
Unknown	1,013 (0.5)	62 (0.2)
Alcohol consumption, median (IQR), g/day	9.0 (0, 26.3)	11.3 (0, 26.8)
Current smoker, n (%)	16,255 (7.8)	1,659 (5.7)
Body mass index, mean (SD), kg/m^2	26.2 (23.7, 29.3)	25.8 (23.4, 28.6)
Total physical activity, MET-mins/week		
0-599	31,022 (14.9)	4,469 (15.2)
600-1199	31,590 (15.1)	4,741 (16.1)
≥1200	109,577 (52.5)	16,023 (54.5)
Unknown	36,529 (17.5)	4,153 (14.1)
Energy intake, median (IQR), kcal/d	1,995.9 (1,672.2, 2,362.6)	2,027.6 (1,718.3, 2,382.7)
Alanine aminotransferase, median (IQR), U/L	19.8 (155.2, 26.8)	19.5 (14.9, 26.4)
Albumin, median (IQR), g/L	45.4 (43.7, 47.1)	45.5 (43.8, 47.1)
Gamma glutamyltransferase, median (IQR), U/L	25.1 (17.9, 38.6)	23.9 (17.3, 36.2)
Dyslipidemia, n (%)	103,544 (49.6)	12,783 (43.5)
Hypertension, n (%)	109,041 (52.2)	13,217 (45.0)
Diabetes, n (%)	10,742 (5.2)	963 (3.3)

Abbreviations: IQR, interquartile range; MET, metabolic equivalent of task; PDFF, proton-density fat fraction; PF, processed food; SD, standard deviation; UPF, ultra-processed food.

Table S6. Associations of UPF and PF intake with MASLD risk*

**UPF, %, median (IQR)**	Quintile 1	Quintile 2	Quintile 3	Quintile 4	Quintile 5	*P* for trend	Per 10% increment
	6.8 (4.6, 8.5)	13.0 (11.6, 14.6)	19.4 (17.8, 21.1)	27.3 (25.0, 29.7)	41.2 (36.3, 48.9)		
No. of participants	5,877	5,877	5,878	5,877	5,877		
No. of cases	1,039	1,126	1,249	1,366	1,591		
Model 1	0 (reference)	1.08 (1.00, 1.17)	1.19 (1.10, 1.28)	1.30 (1.21, 1.39)	1.49 (1.39, 1.59)	<.0001	1.10 (1.09, 1.12)
Model 2	0 (reference)	1.10 (1.02, 1.18)	1.19 (1.10, 1.28)	1.28 (1.19, 1.37)	1.43 (1.34, 1.54)	<.0001	1.09 (1.07, 1.10)
**PF, %, median (IQR)**	Quintile 1	Quintile 2	Quintile 3	Quintile 4	Quintile 5	*P* for trend	Per 10% increment
	3.4 (2.1, 4.5)	7.2 (6.3, 8.1)	11.1 (10.0, 12.2)	16.4 (14.8, 18.2)	28.2 (23.5, 35.9)		
No. of participants	5,877	5,877	5,878	5,877	5,877		
No. of cases	1,236	1,178	1,074	1,244	1,639		
Model 1	0 (reference)	0.94 (0.88, 1.01)	0.84 (0.78, 0.91)	0.92 (0.86, 0.99)	1.08 (1.01, 1.16)	<.0001	1.07 (1.05, 1.09)
Model 2	0 (reference)	0.99 (0.92, 1.06)	0.91 (0.84, 0.99)	0.99 (0.91, 1.08)	1.05 (0.95, 1.15)	0.10	1.05 (1.03, 1.08)

*Data are shown as relative risk (95% CI).

Model 1 was adjusted for age when completing the most recent dietary recall, age when completing the MRI scan, and sex.

Model 2 was adjusted for variables in model 1 and race, education, socioeconomic status, smoking status, alcohol consumption, physical activity, and total energy intake.

Abbreviations: CI, confidence interval; MET, metabolic equivalent of task; PDFF, proton-density fat fraction; PF, processed food; UPF, ultra-processed food.

Table S7. β-coefficients (95%CI) of PDFF and liver cT1 associated with UPF and PF intake

**UPF, %, median**	**Quintile 1**	**Quintile 2**	**Quintile 3**	**Quintile 4**	**Quintile 5**	***P* for trend**	**Per 10% increment**
	6.8	13	19.4	27.3	41.2		
**PDFF, median (IQR)**	2.55 (1.83, 4.06)	2.58 (1.82, 4.21)	2.71 (1.90, 4.49)	2.80 (1.93, 4.82)	3.07 (1.99, 5.47)		
No. of participants	5,877	5,877	5,878	5,877	5,877		
Model 1	0 (reference)	0.15 (0, 0.29)	0.52 (0.37, 0.66)	0.31 (0.16, 0.45)	0.94 (0.79, 1.08)	<.0001	0.25 (0.21, 0.28)
Model 2	0 (reference)	0.18 (0.03, 0.32)	0.32 (0.17, 0.46)	0.50 (0.35, 0.64)	0.86 (0.71, 1.01)	<.0001	0.23 (0.19, 0.26)
**Liver cT1, median (IQR)**	682 (655, 718)	689 (658, 723)	690 (660, 725)	693 (664, 730)	703.5 (671, 742)		
No. of participants	3,768	3,769	3,768	3,769	3,768		
Model 1	0 (reference)	4.56 (2.18, 6.94)	10.59 (8.20, 12.97)	6.40 (4.02, 8.79)	21.13 (18.75, 23.52)	<.0001	5.62 (5.07, 6.16)
Model 2	0 (reference)	2.89 (0.65, 5.13)	3.00 (0.75, 5.26)	5.63 (3.36, 7.90)	11.84 (9.53, 14.15)	0.002	3.17 (2.63, 3.70)
**PF, %, median**	Quintile 1	Quintile 2	Quintile 3	Quintile 4	Quintile 5	*P* for trend	Per 10% increment
	3.4	7.2	11.1	16.4	28.2		
**PDFF, median (IQR)**	2.58 (1.78, 4.45)	2.59 (1.81, 4.27)	2.58 (1.83, 4.11)	2.76 (1.92, 4.51)	3.20 (2.16, 5.57)		
No. of participants	5,877	5,877	5,878	5,877	5,877		
Model 1	0 (reference)	-0.02 (-0.17, 0.13)	-0.28 (-0.42, -0.13)	-0.10 (-0.25, 0.05)	0.36 (0.20, 0.51)	<.0001	0.19 (0.15, 0.24)

Model 2	0 (reference)	0.11 (-0.04, 0.25)	-0.06 (-0.22, 0.09)	0.08 (-0.10, 0.26)	0.32 (0.11, 0.52)	<.0001	0.19 (0.12, 0.25)
**Liver cT1, median (IQR)**	694 (664, 729)	693 (662, 729)	689 (658, 724)	690 (658, 724)	694 (662, 730)		
No. of participants	3,768	3,769	3,768	3,769	3,768		
Model 1	0 (reference)	-1.02 (-3.42, 1.39)	-6.43 (-8.85, -4.01)	-8.28 (-10.73, -5.84)	-8.16 (-10.71, -5.61)	<.0001	-2.05 (-2.82, -1.29)
Model 2	0 (reference)	1.87 (-0.56, 4.30)	0.45 (-2.17, 3.06)	2.52 (-0.39, 5.42)	5.50 (2.10, 8.89)	0.001	2.40 (1.35, 3.45)

Model 1 was adjusted for age when completing the most recent dietary recall, age when completing the MRI scan, and sex.

Model 2 was adjusted for variables in model 1 and race, education, socioeconomic status, smoking status, alcohol consumption, physical activity, and total energy intake.

Abbreviations: CI, confidence interval; IQR; interquartile range; PDFF, proton-density fat fraction; PF, processed food; UPF, ultra-processed food.

Table S8. Stratified analyses by major confounders of association between UPF and PF intake and MASLD risk*

	UPF		PF	
	Per 10% increment	*P*-interaction	Per 10% increment	*P*-interaction
**Age**		0.77		0.34
<60 years	1.10 (1.07, 1.12)		1.06 (1.02, 1.10)	
≥60 years	1.10 (1.06, 1.13)		1.04 (0.99, 1.09)	
**Sex**		0.0068		0.78
Male	1.08 (1.06, 1.11)		1.03 (0.99, 1.07)	
Female	1.12 (1.09, 1.15)		1.13 (1.05, 1.21)	
**Socioeconomic status**		0.21		0.66
<median	1.09 (1.06, 1.11)		1.05 (1.01, 1.10)	
≥median	1.10 (1.07, 1.13)		1.06 (1.01, 1.10)	
**Smoking**		0.027		0.89
Never	1.11 (1.09, 1.14)		1.03 (0.99, 1.08)	
Former or current	1.07 (1.04, 1.10)		1.08 (1.03, 1.12)	
**Physical activity**		0.38		0.31
<1200 MET-mins/week	1.09 (1.06, 1.12)		1.04 (0.99, 1.09)	
≥1200 MET-mins/week	1.11 (1.08, 1.13)		1.06 (1.02, 1.11)	
**Energy Intake**		0.71		0.84
<median	1.10 (1.07, 1.12)		1.21 (1.16, 1.27)	
≥median	1.09 (1.07, 1.12)		1.31 (1.24, 1.38)	

*Data are shown in relative risk (95% CI)

Models were adjusted for age when completing the most recent dietary recall, age when completing the MRI scan, sex, race, education, socioeconomic status, smoking status, alcohol consumption, physical activity, and total energy intake.

Abbreviations: CI, confidence interval; MASLD, metabolic-dysfunction associated steatotic liver disease; MET, metabolic equivalent of task; PF, processed food; UPF, ultra-processed food.

Table S9. Stratified analyses by major confounders of association between UPF and PDFF and liver cT1

UPF intake	PDFF		Liver cT1	
	Per 10% increment	*P*-interaction	Per 10% increment	*P*-interaction
**Age**		0.037		0.67
<60 years	0.24 (0.20, 0.29)		4.60 (3.88, 5.32)	
≥60 years	0.19 (0.14, 0.24)		4.53 (3.65, 5.42)	
**Sex**		0.28		0.074
Male	0.22 (0.17, 0.27)		5.58 (4.75, 6.42)	
Female	0.24 (0.19, 0.28)		3.86 (3.11, 4.61)	
**Socioeconomic status**		0.0094		0.19
<median	0.18 (0.13, 0.23)		4.36 (3.56, 5.16)	
≥median	0.26 (0.21, 0.31)		4.72 (3.94, 5.50)	
**Smoking**		0.29		0.19
Never	0.25 (0.21, 0.29)		4.51 (3.81, 5.20)	
Former or current	0.20 (0.14, 0.26)		4.95 (4.01, 5.89)	
**Physical activity**		0.33		0.92
<1200 MET-mins/week	0.24 (0.17, 0.30)		4.56 (3.56, 5.57)	
≥1200 MET-mins/week	0.22 (0.17, 0.26)		4.63 (3.88, 5.39)	
**Energy Intake**		0.038		0.019
<median	0.19 (0.15, 0.23)		4.05 (3.31, 4.79)	
≥median	0.26 (0.21, 0.31)		5.26 (4.42, 6.11)	

Data are shown in β-coefficient (95% CI).

Models were adjusted for age when completing the most recent dietary recall, age when completing the MRI scan, sex, race, education, socioeconomic status, smoking status, alcohol consumption, physical activity, and total energy intake.

Abbreviations: MET, metabolic equivalent of task; PDFF, proton-density fat fraction; UPF, ultra-processed food.

Table S10. Stratified analyses by major confounders of association between PF and PDFF and liver cT1

PF intake	PDFF		Liver cT1	
	Per 10% increment	*P*-interaction	Per 10% increment	*P*-interaction
**Age**		0.12		0.27
<60 years	0.22 (0.14, 0.31)		3.18 (1.81, 4.55)	
≥60 years	0.12 (0.02, 0.21)		1.06 (-0.56, 2.68)	
**Sex**		0.41		0.03
Male	0.16 (0.08, 0.24)		1.02 (-0.31, 2.35)	
Female	0.21 (0.10, 0.33)		3.15 (1.27, 5.04)	
**Socioeconomic status**		0.85		0.51
<median	0.17 (0.09, 0.26)		1.71 (0.20, 3.21)	
≥median	0.20 (0.11, 0.29)		3.03 (1.57, 4.50)	
**Smoking**		0.0001		0.0003
Never	0.15 (0.07, 0.24)		2.13 (0.71, 3.56)	
Former or current	0.22 (0.12, 0.31)		2.55 (0.98, 4.11)	
**Physical activity**		0.27		0.27
<1200 MET-mins/week	0.23 (0.11, 0.36)		2.79 (0.81, 4.76)	
≥1200 MET-mins/week	0.17 (0.10, 0.25)		2.26 (0.90, 3.63)	
**Energy Intake**		0.83		0.15
<median	0.19 (0.15, 0.23)		3.34 (1.70, 4.99)	
≥median	0.26 (0.21, 0.31)		1.94 (0.57, 3.31)	

Data are shown in β-coefficient (95% CI).

Models were adjusted for age when completing the most recent dietary recall, age when completing the MRI scan, sex, race, education, socioeconomic status, smoking status, alcohol consumption, physical activity, and total energy intake.

Abbreviations: MET, metabolic equivalent of task; PDFF, proton-density fat fraction; PF, processed food.

Table S11. Sensitivity analysis of further adjusting for BMI, baseline chronic diseases, and baseline liver function in the model

	Quintile 1	Quintile 2	Quintile 3	Quintile 4	Quintile 5	*P* for trend	Per 10% increment
**Further adjusted for BMI**							
**UPF**							
MASLD	1 (reference)	1.04 (0.96, 1.13)	1.09 (1.00, 1.19)	1.13 (1.04, 1.23)	1.20 (1.11, 1.30)	<.0001	1.04 (1.03, 1.06)
PDFF	0 (reference)	0.07 (-0.07, 0.2)	0.12 (-0.02, 0.26)	0.22 (0.08, 0.36)	0.43 (0.29, 0.57)	<.0001	0.12 (0.08, 0.15)
Liver cT1	0 (reference)	2.7 (0.46, 4.94)	3.03 (0.78, 5.28)	5.55 (3.28, 7.81)	11.68 (9.38, 13.99)	<.0001	3.14 (2.61, 3.67)
**PF**							
MASLD	1 (reference)	0.998 (0.92, 1.08)	0.93 (0.85, 1.02)	1.01 (0.92, 1.11)	1.03 (0.92, 1.15)	0.39	1.04 (1.01, 1.07)
PDFF	0 (reference)	0.12 (-0.02, 0.26)	-0.01 (-0.16, 0.14)	0.14 (-0.03, 0.30)	0.29 (0.10, 0.49)	0.0031	0.16 (0.10, 0.22)
Liver cT1	0 (reference)	2.07 (-0.22, 4.36)	0.91 (-1.56, 3.37)	2.81 (0.08, 5.55)	5.13 (1.93, 8.32)	0.0021	1.95 (0.96, 2.93)
**Further adjusted for baseline chronic disease**							
**UPF**							
MASLD	1 (reference)	1.04 (0.96, 1.13)	1.09 (0.999, 1.18)	1.13 (1.04, 1.23)	1.19 (1.10, 1.29)	<.0001	1.04 (1.03, 1.06)
PDFF	0 (reference)	0.13 (-0.01, 0.27)	0.23 (0.09, 0.37)	0.39 (0.25, 0.53)	0.69 (0.55, 0.83)	<.0001	0.19 (0.15, 0.22)
Liver cT1	0 (reference)	3.23 (0.95, 5.52)	4.32 (2.02, 6.62)	7.65 (5.34, 9.96)	15.01 (12.67, 17.36)	<.0001	4.11 (3.57, 4.65)

**PF**							
MASLD	1 (reference)	0.99 (0.91, 1.07)	0.93 (0.85, 1.02)	1.00 (0.91, 1.1)	1.02 (0.91, 1.13)	0.57	1.04 (1.01, 1.07)
PDFF	0 (reference)	0.10 (-0.05, 0.24)	-0.04 (-0.20, 0.11)	0.09 (-0.08, 0.26)	0.28 (0.08, 0.48)	0.0034	0.16 (0.10, 0.22)
Liver cT1	0 (reference)	1.38 (-0.97, 3.72)	0.28 (-2.24, 2.8)	1.85 (-0.95, 4.64)	4.40 (1.13, 7.67)	0.0071	1.85 (0.84, 2.86)
**Further adjusted for baseline liver function**							
**UPF**							
MASLD	1 (reference)	1.04 (0.95, 1.13)	1.09 (1.00, 1.18)	1.12 (1.03, 1.21)	1.19 (1.09, 1.29)	<.0001	1.04 (1.02, 1.06)
PDFF	0 (reference)	0.12 (-0.02, 0.26)	0.25 (0.12, 0.39)	0.40 (0.26, 0.53)	0.69 (0.55, 0.83)	<.0001	0.19 (0.16, 0.22)
Liver cT1	0 (reference)	3.34 (1.05, 5.62)	4.68 (2.38, 6.98)	7.36 (5.05, 9.67)	15.33 (12.98, 17.67)	<.0001	4.12 (3.58, 4.66)
**PF**							
MASLD	1 (reference)	0.99 (0.92, 1.08)	0.93 (0.85, 1.02)	1.004 (0.91, 1.11)	1.03 (0.92, 1.15)	0.41	1.04 (1.01, 1.07)
PDFF	0 (reference)	0.11 (-0.03, 0.25)	-0.07 (-0.22, 0.08)	0.06 (-0.11, 0.23)	0.27 (0.08, 0.47)	0.0044	0.15 (0.09, 0.21)
Liver cT1	0 (reference)	2.02 (-0.32, 4.36)	0.51 (-2.01, 3.03)	2.28 (-0.52, 5.08)	4.77 (1.50, 8.03)	0.005	1.88 (0.87, 2.90)
**Further excluding participants with excess alcohol intake***							
**UPF**							

MASLD	1 (reference)	1.14 (1.03, 1.26)	1.28 (1.16, 1.41)	1.40 (1.27, 1.54)	1.58 (1.45, 1.73)	<.0001	1.09 (1.07, 1.10)
PDFF	0 (reference)	0.25 (0.09, 0.42)	0.38 (0.21, 0.54)	0.55 (0.38, 0.72)	1.03 (0.86, 1.20)	<.0001	0.25 (0.21, 0.29)
Liver cT1	0 (reference)	2.84 (0.05, 5.62)	5.04 (2.23, 7.85)	8.13 (5.32, 10.95)	18.53 (15.67, 21.38)	<.0001	4.85 (4.21, 5.48)
**PF**							
MASLD	1 (reference)	0.98 (0.90, 1.06)	0.96 (0.89, 1.05)	0.92 (0.84, 1.01)	0.99 (0.90, 1.09)	0.73	0.98 (0.93, 1.03)
PDFF	0 (reference)	0.01 (-0.16, 0.18)	0.03 (-0.14, 0.21)	-0.04 (-0.23, 0.14)	0.15 (-0.06, 0.35)	0.21	0.05 (-0.05, 0.15)
Liver cT1	0 (reference)	-0.28 (-3.12, 2.57)	1.32 (-1.61, 4.24)	0.91 (-2.15, 3.97)	2.77 (-0.63, 6.17)	0.085	2.38 (0.66, 4.1)

The effect size for MASLD was shown in relative risk (95% CI) and the effect sizes for PDFF and liver cT1 were shown in β-coefficient (95% CI).

Models were adjusted for age when completing the most recent dietary recall, age when completing the MRI scan, sex, race, education, socioeconomic status, smoking status, alcohol consumption, physical activity, and total energy intake.

*: >30 g/day for men and >20 g/day for women.

Abbreviations: CI, confidence interval; MET, metabolic equivalent of task; PDFF, proton-density fat fraction; PF, processed food; UPF, ultra-processed food.

Table S12. Sensitivity analysis of Propensity Score Matching

	Ultra-processed food				
	Quintile 1	Quintile 2	Quintile 3	Quintile 4	Quintile 5
MASLD	1.00 (reference)	1.02 (0.95, 1.10)	1.06 (0.99, 1.14)	1.09 (1.02, 1.17)	1.18 (1.10, 1.26)
PDFF	1.00 (reference)	0.04 (-0.09, 0.17)	0.10 (-0.04, 0.23)	0.20 (0.06, 0.34)	0.45 (0.29, 0.60)
Liver Ct1	1.00 (reference)	3.50 (1.19, 5.81)	4.42 (2.11, 6.73)	7.43 (5.04, 9.82)	15.9 (13.3, 18.5)

The effect size for MASLD was shown in relative risk (95% CI) and the effect sizes for PDFF and liver cT1 were shown in β-coefficient (95% CI).

The Propensity Score Matching was included age when completing the most recent dietary recall, age when completing the MRI scan, sex, education, socioeconomic status, physical activity, and BMI.

Abbreviations: CI, confidence interval; MET, metabolic equivalent of task; PDFF, proton-density fat fraction; PF, processed food; UPF, ultra-processed food.

Table S13. The mediation of BMI in the association of UPF with MASLD risk, PDFF and liver cT1

	Direct Effect (95% CI)	Indirect Effect (95% CI)	Mediation proportion, %
**MASLD***	1.46 (1.21, 1.72)	1.57 (1.50, 1.64)	64.33 (55.07, 73.59)
**PDFF****	0.96 (0.64, 1.28)	1.26 (1.14, 1.39)	56.90 (48.28, 65.51)
**Liver cT1****	28.53 (23.23, 33.83)	17.40 (15.38, 19.41)	37.88 (32.62, 43.13)

*Effect shown in relative risk (95% CI); **Effect shown in β-coefficient (95% CI).

Models were adjusted for age when completing the most recent dietary recall, age when completing the MRI scan, sex, race, education, socioeconomic status, smoking status, alcohol consumption, physical activity, and total energy intake.

Abbreviations: CI, confidence interval; MASLD, metabolic-dysfunction associated steatotic liver disease; PDFF, proton-density fat fraction; UPF, ultra-processed food.

Table S14. The association of 283 plasma proteins with UPF intake and the mediation in UPF-PDFF association

Protein	β of UPF	*p*_value	q_value	proportion	LCI	UCI	*p*_value*	q_value#
FGF21	0.756951	1.05E-07	4.95E-06	0.393517	0.192553	1.149133	<0.0001	<0.0001
GSTA3	0.42454	7.14E-06	0.000119	0.381708	0.17596	0.975444	<0.0001	<0.0001
HNMT	0.233757	2.49E-05	0.000359	0.260611	0.115481	0.773686	<0.0001	<0.0001
TNFRSF10B	0.181363	5.36E-05	0.00066	0.096931	0.033958	0.307721	<0.0001	<0.0001
IDUA	0.227648	7.08E-05	0.000835	0.220178	0.092355	0.676458	<0.0001	<0.0001
CDHR5	0.333001	2.31E-11	3.27E-09	0.368916	0.187825	1.29008	0.002	0.029789
FGFR2	0.129333	3.50E-07	1.41E-05	0.278354	0.126506	0.881349	0.002	0.029789
IL18R1	0.202148	5.41E-07	1.91E-05	0.354648	0.170227	1.104874	0.002	0.029789
GFRA1	0.154538	6.88E-07	1.95E-05	0.251189	0.120677	0.772349	0.002	0.029789
PAMR1	0.171307	1.43E-06	3.68E-05	0.329498	0.14999	1.047497	0.002	0.029789
COL4A1	-0.22726	2.05E-06	4.83E-05	0.227488	0.090809	0.803525	0.002	0.029789
NTRK3	-0.12149	6.22E-06	0.00011	0.221285	0.092374	0.750048	0.002	0.029789
CCL22	0.260194	3.31E-05	0.000426	0.161487	0.062845	0.523347	0.002	0.029789
PGF	0.113936	8.58E-05	0.000972	0.147953	0.054144	0.477654	0.002	0.029789
CTSO	0.136369	0.000201	0.002083	0.283285	0.122999	0.883314	0.002	0.029789
PDGFC	0.124355	0.000422	0.003722	0.123146	0.042223	0.351728	0.002	0.029789
CNTN5	-0.15223	0.000518	0.004093	0.105254	0.037206	0.357086	0.002	0.029789
CA9	-0.1721	0.000727	0.005373	0.165941	0.069857	0.524405	0.002	0.029789
PXN	0.191578	0.00318	0.017744	0.078376	0.023714	0.253782	0.002	0.029789
FABP4	0.483108	6.02E-12	1.70E-09	0.683443	0.378712	2.543631	0.004	0.032343
VWC2	0.264532	2.53E-09	2.39E-07	0.08715	0.026083	0.282961	0.004	0.032343
IL1RN	0.386348	1.54E-08	1.09E-06	0.517751	0.284053	1.523797	0.004	0.032343
CPM	0.242987	3.33E-08	1.88E-06	0.518238	0.270157	1.687252	0.004	0.032343
RBP5	0.34052	6.84E-07	1.95E-05	0.458389	0.217628	1.355398	0.004	0.032343
CREG1	0.214607	3.85E-06	8.38E-05	0.400534	0.186295	1.386862	0.004	0.032343
GUSB	0.339028	4.58E-06	9.27E-05	0.559344	0.276529	1.916183	0.004	0.032343
SELE	0.285021	5.31E-06	0.0001	0.326922	0.145671	1.207811	0.004	0.032343

Table S14 continued

Protein	β of UPF	*p*_value	q_value	proportion	LCI	UCI	*p*_value	q_value
BPIFB2	0.377237	8.00E-06	0.000126	0.416007	0.178094	1.559813	0.004	0.032343
PLAT	0.235529	0.000213	0.002083	0.417798	0.186292	1.542194	0.004	0.032343
NMNAT1	0.320026	0.000252	0.00238	0.062498	0.016056	0.201495	0.004	0.032343
NADK	0.238974	0.000336	0.003064	0.138987	0.049108	0.474064	0.004	0.032343
CA14	-0.15248	0.000472	0.003931	0.247532	0.097128	0.738778	0.004	0.032343
SIGLEC7	0.117474	0.000521	0.004093	0.176285	0.057694	0.624012	0.004	0.032343
IL13RA1	0.088274	0.001149	0.007561	0.191205	0.066495	0.538684	0.004	0.032343
C19orf12	0.237579	0.002021	0.011672	0.233058	0.079824	0.694851	0.004	0.032343
CES1	0.406499	2.54E-05	0.000359	0.445621	0.169305	1.646073	0.006	0.043538
KRT18	0.360679	0.000207	0.002083	0.357266	0.129914	1.036372	0.006	0.043538
FABP3	0.210976	0.000971	0.006542	0.193747	0.063553	0.726234	0.006	0.043538
FABP5	0.240645	0.004304	0.022676	0.138555	0.041341	0.461643	0.006	0.043538
SSC5D	0.200285	0.000213	0.002083	0.280428	0.090633	1.11216	0.008	0.049217
CD8A	0.204003	0.000591	0.004519	0.083883	0.02238	0.289089	0.008	0.049217
ERN1	0.163155	0.001359	0.008361	0.157067	0.037097	0.610158	0.008	0.049217
CDHR2	0.302375	0.001745	0.01051	0.331932	0.105525	0.936497	0.008	0.049217
SCARB2	0.09029	0.005021	0.024082	0.131879	0.030278	0.429562	0.008	0.049217
REN	0.191861	0.006852	0.030453	0.091593	0.024128	0.32504	0.008	0.049217
TREM2	0.113434	0.059488	0.175136	0.119024	0.027495	0.360246	0.008	0.049217
GUCA2A	-0.10975	0.003367	0.018327	0.13827	0.044861	0.487589	0.01	0.057755
ACP5	0.110197	0.006738	0.030453	0.143675	0.033219	0.535503	0.01	0.057755
SDC4	0.138172	0.011481	0.045762	0.070095	0.01496	0.257665	0.01	0.057755
PTS	0.176001	0.007528	0.032277	0.216541	0.04439	0.643131	0.012	0.06792
WFIKKN2	-0.1442	0.000759	0.005373	0.181971	0.055333	0.556506	0.014	0.076192
IGSF3	0.111848	0.006496	0.030135	0.242863	0.048794	0.749413	0.014	0.076192
PTP4A3	0.238257	0.000434	0.003722	0.201472	0.049771	1.083228	0.016	0.082327
GRPEL1	0.194009	0.006887	0.030453	0.091007	0.016159	0.303395	0.016	0.082327

Table S14 continued

Protein	β of UPF	*p*_value	q_value	proportion	LCI	UCI	*p*_value	q_value
MERTK	0.046288	0.118912	0.268043	-0.04011	-0.17136	-0.00602	0.016	0.082327
PRG2	0.2046	0.001274	0.008012	-0.08101	-0.46984	-0.01276	0.018	0.087828
NCF2	0.299507	0.004327	0.022676	0.043262	0.006342	0.142174	0.018	0.087828
TREH	0.178702	0.070886	0.198622	0.148924	0.025492	0.609191	0.018	0.087828
ERBB2	0.067782	0.025397	0.095832	0.23162	0.040223	0.712806	0.02	0.095932
ERBB4	-0.07874	0.005488	0.025885	0.060352	0.010866	0.215221	0.022	0.098825
ACY1	0.200376	0.007059	0.030734	0.305294	0.053166	0.909165	0.022	0.098825
NPTX1	-0.10237	0.032117	0.113614	0.038237	0.004541	0.146703	0.022	0.098825
SMPDL3A	0.151937	0.054534	0.167753	0.042686	0.005309	0.142634	0.022	0.098825
CSF3R	0.127131	0.001194	0.00768	0.058335	0.006277	0.318518	0.024	0.104492
IGFBP2	-0.20826	0.01167	0.045868	0.348092	0.088436	1.144541	0.024	0.104492
CLEC4A	-0.12291	0.004572	0.023103	0.087939	0.013945	0.286948	0.026	0.108206
SCARA5	0.090345	0.007826	0.033054	0.104014	0.014241	0.358754	0.026	0.108206
PTN	0.152123	0.010582	0.042783	0.039978	0.004146	0.167639	0.026	0.108206
CNTN2	-0.11373	0.03837	0.127749	0.035629	0.003657	0.133239	0.028	0.114841
GAS6	-0.09369	0.001801	0.010619	-0.0389	-0.21509	-0.00393	0.03	0.121286
TNFRSF11B	0.060274	0.080296	0.208475	0.041967	0.001789	0.143397	0.044	0.17538
ADIPOQ	-0.18362	0.036435	0.122751	0.244546	0.003516	1.03955	0.046	0.180806
CRELD2	0.16325	0.009059	0.037156	0.036551	0.000937	0.127514	0.048	0.183568
CCL2	0.119136	0.060029	0.175136	0.028574	0.000828	0.103255	0.048	0.183568
ADGRF5	0.103547	0.004752	0.023187	0.037625	########	0.169286	0.052	0.196213
EPHA10	0.145117	0.073511	0.198938	-0.02455	-0.11019	0.000118	0.054	0.201079
TCTN3	0.10249	0.008458	0.035202	0.156618	-0.00561	0.628272	0.058	0.213169
PIGR	0.087924	0.03417	0.11763	0.071967	-0.00323	0.260853	0.066	0.23643
ICAM4	-0.12939	0.043291	0.140021	0.025295	-0.00163	0.099592	0.066	0.23643
CD200	-0.06904	0.028688	0.105437	0.091466	-0.01137	0.31793	0.074	0.261775
WNT9A	0.029545	0.402634	0.596573	-0.06452	-0.28723	0.005766	0.078	0.272519

Table S14 continued								
Protein	β of UPF	*p*_value	q_value	proportion	LCI	UCI	*p*_value	q_value
IL1R1	-0.03491	0.173815	0.339239	0.058766	-0.0076	0.232797	0.082	0.283
PLIN1	0.075236	0.094093	0.229554	0.096222	-0.01245	0.32756	0.09	0.303214
TMPRSS5	-0.07362	0.119906	0.268043	0.055413	-0.01128	0.205757	0.09	0.303214
IDI2	0.106766	0.059447	0.175136	0.022211	-0.00363	0.089948	0.11	0.366235
IL22	0.17637	0.04354	0.140021	0.025925	-0.00486	0.11819	0.118	0.383839
TG	0.199176	0.162074	0.325571	-0.05125	-0.29242	0.010234	0.118	0.383839
MAN2B2	0.111306	0.147098	0.300105	0.123071	-0.03196	0.518538	0.128	0.411636
GPRC5C	0.164194	0.01995	0.076294	0.041354	-0.01121	0.190807	0.136	0.427644
RAD23B	0.052732	0.360538	0.566675	0.020536	-0.00487	0.082801	0.136	0.427644
KLB	-0.19754	0.03064	0.110211	0.061004	-0.02202	0.23433	0.144	0.447824
LRIG1	0.119153	0.003198	0.017744	-0.01911	-0.09431	0.005349	0.146	0.449109
APOF	-0.06933	0.034212	0.11763	0.178837	-0.09234	0.699612	0.148	0.450366
LRTM1	-0.08774	0.109539	0.256194	0.022881	-0.00917	0.137293	0.158	0.475681
SULT2A1	0.142282	0.02588	0.096371	0.108101	-0.03657	0.357644	0.16	0.476632
HSD17B14	0.133055	0.103452	0.245543	0.054853	-0.02066	0.287127	0.168	0.49525
IGDCC3	0.106512	0.104117	0.245543	0.027935	-0.01355	0.149326	0.176	0.513485
TNFRSF13B	0.072062	0.052938	0.164632	0.015041	-0.00539	0.071435	0.182	0.525571
SIGLEC6	0.109981	0.004471	0.023005	0.013798	-0.0079	0.06653	0.186	0.531697
ROR1	0.043331	0.22396	0.403698	-0.05667	-0.28976	0.025323	0.188	0.53204
KLK11	0.042946	0.241736	0.417143	-0.01821	-0.09977	0.009089	0.19	0.532376
CCL17	0.155657	0.16221	0.325571	0.035574	-0.01729	0.149644	0.196	0.538524
NEFL	0.036546	0.466161	0.649514	-0.03462	-0.17156	0.017726	0.196	0.538524
PKD1	-0.05719	0.087214	0.220372	0.019884	-0.01049	0.10911	0.198	0.538788
CXCL17	0.06321	0.214104	0.396022	0.021751	-0.01142	0.095707	0.2	0.539048
COL9A1	0.085233	0.093276	0.22954	-0.01472	-0.07801	0.00801	0.202	0.539302
PHLDB2	0.125411	0.306701	0.49598	0.024354	-0.01549	0.1617	0.208	0.550131
APOL1	0.16026	0.045262	0.143922	0.023319	-0.01623	0.130868	0.21	0.550278

Table S14 continued								
Protein	β of UPF	*p*_value	q_value	proportion	LCI	UCI	*p*_value	q_value
IL12RB1	0.087464	0.057177	0.17399	0.012154	-0.00713	0.064884	0.22	0.571009
PSMA1	0.108149	0.07611	0.201301	0.01125	-0.00947	0.055954	0.228	0.571009
GH1	-0.38693	0.092062	0.228737	0.075751	-0.05985	0.333355	0.226	0.571009
AGT	0.038179	0.219135	0.397985	0.017574	-0.01085	0.095235	0.222	0.571009
GORASP2	0.077058	0.219384	0.397985	0.041749	-0.03312	0.225273	0.228	0.571009
FAM3C	0.112423	0.000755	0.005373	0.017748	-0.01335	0.085812	0.242	0.575513
DHPS	0.111026	0.030766	0.110211	0.040442	-0.03064	0.201843	0.236	0.575513
PBXIP1	0.08185	0.046535	0.146327	0.037557	-0.03742	0.204546	0.242	0.575513
CHRDL2	-0.1029	0.124091	0.269417	-0.03039	-0.19306	0.021565	0.236	0.575513
ACOX1	0.067864	0.260541	0.444176	0.012413	-0.00967	0.070777	0.242	0.575513
IL1R2	0.017778	0.450052	0.642437	0.050899	-0.034	0.200307	0.232	0.575513
ANGPT2	-0.01123	0.806325	0.88349	0.018811	-0.01417	0.087148	0.254	0.599017
VAMP5	0.076127	0.232701	0.407634	0.011215	-0.01118	0.067218	0.26	0.603115
MNAT1	-0.02361	0.672195	0.79628	-0.01452	-0.13193	0.020753	0.26	0.603115
PAGR1	0.077361	0.165083	0.329004	0.027892	-0.02514	0.142083	0.266	0.611645
DPP10	-0.03318	0.408973	0.600874	0.018213	-0.01693	0.088971	0.268	0.611645
CD3G	-0.22602	0.070279	0.198622	-0.0132	-0.1362	0.014442	0.282	0.628394
LPL	-0.07727	0.182129	0.353031	0.088976	-0.11092	0.396953	0.282	0.628394
CDH15	0.061747	0.364981	0.566675	0.064111	-0.06378	0.270778	0.28	0.628394
AGER	-0.05744	0.229285	0.407634	0.040021	-0.04569	0.184566	0.286	0.632328
DLL1	0.080823	0.012706	0.049259	-0.01286	-0.07587	0.01093	0.296	0.643771
FUOM	0.146929	0.07374	0.198938	0.128049	-0.16874	0.598538	0.296	0.643771
SLC39A14	0.011402	0.773181	0.867759	-0.05272	-0.27377	0.044419	0.298	0.643771
HS6ST1	0.075818	0.068792	0.196647	0.02311	-0.02644	0.111611	0.308	0.650478
CD36	0.096207	0.111658	0.25901	0.041854	-0.0702	0.264202	0.308	0.650478
LEO1	0.030507	0.402606	0.596573	0.041101	-0.05531	0.234283	0.306	0.650478
NCAN	-0.08346	0.073172	0.198938	0.044867	-0.0596	0.227865	0.312	0.652759

Table S14 continued								
Protein	β of UPF	*p*_value	q_value	proportion	LCI	UCI	*p*_value	q_value
VCPKMT	0.145407	0.206557	0.384577	0.017124	-0.02949	0.123524	0.316	0.652759
CBLN4	-0.03867	0.317224	0.509322	-0.04437	-0.2594	0.046951	0.316	0.652759
TAGLN3	-0.00813	0.919186	0.932299	0.01489	-0.02346	0.1104	0.32	0.656232
ENPP5	-0.04249	0.435514	0.632054	0.011046	-0.01533	0.067694	0.326	0.663727
VNN2	0.036603	0.520159	0.687874	0.010838	-0.01577	0.063007	0.338	0.678397
MTUS1	-0.01747	0.808565	0.88349	-0.04521	-0.40574	0.065733	0.336	0.678397
IKZF2	0.179232	0.13737	0.292062	-0.00884	-0.05942	0.011325	0.346	0.687007
FGF20	0.054532	0.372876	0.5735	-0.01627	-0.16733	0.025856	0.352	0.687007
IL34	0.034366	0.454019	0.642437	0.007386	-0.00969	0.048554	0.35	0.687007
INPP5J	0.019169	0.718208	0.826231	-0.01307	-0.11665	0.019551	0.348	0.687007
ADA2	0.076093	0.112927	0.259823	0.008978	-0.01638	0.06877	0.364	0.696027
UROS	-0.14109	0.125339	0.269417	-0.01007	-0.09894	0.01887	0.364	0.696027
DDA1	0.035041	0.480018	0.656755	-0.02474	-0.21358	0.041059	0.36	0.696027
GAMT	0.056215	0.238431	0.413963	-0.01079	-0.12893	0.014088	0.368	0.698953
AGR2	-0.03534	0.771862	0.867759	0.00973	-0.01609	0.063396	0.374	0.705613
DEFB116	-0.15029	0.147401	0.300105	-0.0083	-0.09223	0.015431	0.382	0.715934
PTPRF	0.036168	0.303559	0.493719	-0.01042	-0.0777	0.01545	0.39	0.726118
CHI3L1	0.098797	0.249004	0.42708	0.035276	-0.06153	0.201678	0.396	0.732471
CPXM2	0.021234	0.493281	0.664755	0.026172	-0.04775	0.156214	0.404	0.742416
KLK3	0.180025	0.100949	0.242111	-0.00788	-0.08342	0.013658	0.408	0.744929
MORN4	-0.17953	0.085183	0.217179	-0.00832	-0.10963	0.018164	0.416	0.754667
PRSS53	-0.05198	0.300062	0.493707	-0.01458	-0.1409	0.028245	0.426	0.763025
MATN2	0.034747	0.318551	0.509322	0.021326	-0.03499	0.106508	0.426	0.763025
LAMB1	0.080801	0.039993	0.131606	0.022995	-0.04991	0.142733	0.436	0.776025
AHSG	0.058152	0.100951	0.242111	0.041147	-0.09073	0.247359	0.446	0.786111
PON1	-0.0481	0.139323	0.292062	-0.00578	-0.07463	0.015406	0.45	0.786111
SCT	-0.05941	0.30343	0.493719	0.019583	-0.05227	0.149958	0.45	0.786111

Table S14 continued

Protein	β of UPF	*p*_value	q_value	proportion	LCI	UCI	*p*_value	q_value
PPIE	0.10168	0.202829	0.380137	0.013278	-0.0433	0.116466	0.456	0.786878
IGFBPL1	0.01517	0.684877	0.797779	0.03239	-0.07288	0.176662	0.456	0.786878
IL6R	0.036935	0.379514	0.580554	0.015583	-0.04636	0.116831	0.462	0.787627
CPA4	0.014452	0.793176	0.88027	0.009824	-0.02842	0.082169	0.46	0.787627
PRSS27	0.035625	0.460501	0.645157	-0.0062	-0.05882	0.01297	0.474	0.789071
FCGR2B	0.047335	0.491418	0.664755	0.028473	-0.06344	0.141201	0.472	0.789071
WAS	-0.03694	0.663808	0.796007	-0.0048	-0.05308	0.011823	0.474	0.789071
RGS8	0.01265	0.877956	0.912577	0.005075	-0.01079	0.042147	0.468	0.789071
DCBLD2	0.059175	0.145362	0.300105	-0.02202	-0.1552	0.047691	0.492	0.814246
GFRAL	0.10536	0.197714	0.373021	-0.00985	-0.10394	0.029898	0.5	0.817919
CD14	0.049897	0.29487	0.488002	-0.0056	-0.0676	0.011443	0.5	0.817919
MET	-0.04608	0.05832	0.175136	0.05939	-0.24236	0.360129	0.516	0.839241
RECK	-0.004	0.882279	0.912577	0.009098	-0.02948	0.079131	0.52	0.840914
RASGRF1	-0.09085	0.125664	0.269417	0.008164	-0.02211	0.086266	0.528	0.849
BRD3	0.092229	0.120288	0.268043	-0.00865	-0.10927	0.021588	0.544	0.869785
ITGB5	-0.02469	0.591781	0.74279	-0.00294	-0.04542	0.010126	0.552	0.877618
GART	0.121823	0.000787	0.005431	0.009881	-0.04181	0.082584	0.578	0.889005
KIR3DL1	-0.26869	0.073811	0.198938	0.006598	-0.02247	0.051352	0.58	0.889005
PDIA5	-0.14361	0.082656	0.212652	-0.00488	-0.0839	0.023476	0.574	0.889005
BPIFB1	-0.09563	0.2263	0.405335	0.029696	-0.14072	0.237097	0.598	0.889005
CCL25	-0.06704	0.269236	0.45625	0.005025	-0.01884	0.046469	0.582	0.889005
SDK2	0.051672	0.288766	0.483554	0.003778	-0.02209	0.053493	0.596	0.889005
PROCR	-0.03476	0.459733	0.645157	0.00466	-0.02481	0.073012	0.598	0.889005
PTH	0.071638	0.492981	0.664755	-0.02294	-0.25686	0.085434	0.596	0.889005
FGFBP1	-0.02277	0.541483	0.702934	0.015293	-0.05672	0.11163	0.572	0.889005
JAM2	0.017219	0.539209	0.702934	-0.00346	-0.04462	0.013156	0.576	0.889005
HLA-A	0.016357	0.701638	0.813785	-0.004	-0.06606	0.019976	0.6	0.889005

Table S14 continued

Protein	β of UPF	*p*_value	q_value	proportion	LCI	UCI	*p*_value	q_value
STX3	-0.01653	0.74653	0.848466	-0.00864	-0.11107	0.038003	0.596	0.889005
IL17RB	-0.00385	0.952886	0.952886	-0.03366	-0.25143	0.091108	0.598	0.889005
EPPK1	0.201299	0.034499	0.11763	0.005423	-0.02622	0.056172	0.608	0.891523
SSH3	0.047952	0.215771	0.396513	0.005381	-0.03462	0.06984	0.606	0.891523
FAM171A2	-0.04999	0.808114	0.88349	-0.00469	-0.07688	0.022915	0.612	0.892763
LTBP2	-0.04731	0.195085	0.370531	0.024245	-0.12173	0.194164	0.624	0.899279
MATN3	-0.03662	0.326478	0.519064	0.018239	-0.07704	0.129825	0.626	0.899279
CLNS1A	-0.03803	0.39932	0.596573	0.003649	-0.02146	0.057257	0.624	0.899279
LELP1	-0.01281	0.893703	0.916034	0.004318	-0.02819	0.053221	0.632	0.903313
CACNA1H	-0.06832	0.388792	0.588386	0.004362	-0.03358	0.076717	0.638	0.907307
KIT	-0.05451	0.123475	0.269417	0.021622	-0.12241	0.198013	0.644	0.91126
CXADR	-0.04676	0.446694	0.641697	-0.00364	-0.0465	0.020791	0.654	0.916248
SERPINB5	0.013854	0.82246	0.888382	-0.00228	-0.04425	0.013968	0.654	0.916248
GUK1	0.045303	0.446378	0.641697	0.004749	-0.0349	0.067404	0.666	0.928463
UNG	0.085143	0.078388	0.205405	0.006239	-0.04527	0.071599	0.672	0.932235
S100A13	0.026263	0.580011	0.736067	0.004235	-0.04429	0.062359	0.678	0.935971
SCARB1	-0.08507	0.233345	0.407634	-0.00152	-0.03706	0.01108	0.69	0.947913
PPP1R12B	0.150106	0.125311	0.269417	0.002607	-0.02825	0.050082	0.704	0.951419
ART5	0.109991	0.166717	0.329937	-0.00282	-0.05862	0.016407	0.706	0.951419
EGFLAM	0.009137	0.814825	0.883508	0.00203	-0.02674	0.041483	0.696	0.951419
FUCA1	0.019414	0.848227	0.895702	0.005768	-0.04539	0.065683	0.706	0.951419
SIRPB1	0.150339	0.004737	0.023187	0.003018	-0.02578	0.041039	0.724	0.952017
LIFR	-0.05053	0.092141	0.228737	0.002252	-0.02409	0.039982	0.77	0.952017
IGFBP1	-0.23926	0.139184	0.292062	0.039982	-0.38382	0.332399	0.714	0.952017
MDGA1	-0.12532	0.140852	0.293096	0.001763	-0.0217	0.036909	0.792	0.952017
IFIT3	0.064727	0.168519	0.331186	0.001441	-0.0313	0.045052	0.772	0.952017
NPY	0.103703	0.193325	0.369669	0.008527	-0.07078	0.0849	0.766	0.952017

Table S14 continued								
Protein	β of UPF	*p*_value	q_value	proportion	LCI	UCI	*p*_value	q_value
CD207	0.049307	0.285291	0.480579	-0.00177	-0.03837	0.019692	0.762	0.952017
TGFBR1	-0.02897	0.366436	0.566675	0.002214	-0.02463	0.061704	0.728	0.952017
APOB	0.023506	0.428315	0.62481	0.003589	-0.05135	0.064818	0.794	0.952017
SPTLC1	0.03242	0.478365	0.656755	0.006846	-0.07679	0.091988	0.766	0.952017
FCRL5	0.049145	0.480382	0.656755	0.006606	-0.07233	0.07964	0.788	0.952017
NECTIN1	0.027771	0.510518	0.681494	0.001293	-0.02924	0.03863	0.79	0.952017
KAZALD1	0.035819	0.514297	0.683315	-0.00116	-0.03081	0.012667	0.784	0.952017
TGOLN2	0.019858	0.526983	0.693657	0.001112	-0.02099	0.033149	0.79	0.952017
L1CAM	-0.02155	0.531847	0.696818	0.000978	-0.01872	0.033086	0.804	0.952017
NCAM1	-0.02143	0.564444	0.729395	0.007904	-0.08832	0.11499	0.784	0.952017
DENND2B	0.032496	0.593182	0.74279	-0.00232	-0.07388	0.038205	0.802	0.952017
ENSA	0.034599	0.62346	0.760514	0.007707	-0.06545	0.088403	0.728	0.952017
MORC3	-0.01529	0.670284	0.79628	-0.00191	-0.05674	0.018236	0.714	0.952017
DNAJC21	0.021568	0.67962	0.797779	0.007921	-0.08259	0.09871	0.742	0.952017
MYO9B	0.031757	0.712636	0.823167	0.001992	-0.03025	0.036175	0.802	0.952017
NTproBNP	-0.03523	0.745202	0.848466	-0.01397	-0.21317	0.110295	0.776	0.952017
KLK15	0.020776	0.760746	0.861164	0.003777	-0.03743	0.055269	0.718	0.952017
IVD	-0.01341	0.834913	0.89322	-0.00083	-0.02832	0.015753	0.798	0.952017
GSN	0.003472	0.883555	0.912577	-0.01208	-0.18691	0.107312	0.774	0.952017
CALY	0.00677	0.889987	0.915878	0.001209	-0.02972	0.059077	0.804	0.952017
KRT14	-0.0048	0.896613	0.916034	-0.00099	-0.02696	0.010202	0.782	0.952017
TMEM25	0.005819	0.922416	0.932299	-0.00678	-0.099	0.054307	0.758	0.952017
SCGB2A2	-0.00294	0.938789	0.942118	-0.00129	-0.05724	0.029577	0.792	0.952017
HLA-DRA	0.047218	0.292417	0.486789	-0.00077	-0.02451	0.011898	0.816	0.9622
TMOD4	-0.02764	0.620088	0.759674	-0.00145	-0.06129	0.03742	0.82	0.962905
NGF	-0.00501	0.743093	0.848466	-0.00079	-0.03532	0.018925	0.826	0.965942
ADAMTSL2	0.07298	0.063238	0.182616	-0.01942	-0.51721	0.200375	0.84	0.969304

Table S14 continued								
Protein	β of UPF	*p*_value	q_value	proportion	LCI	UCI	*p*_value	q_value
SHH	-0.03289	0.578395	0.736067	0.000766	-0.02905	0.041237	0.84	0.969304
FHIP2A	-0.03571	0.578244	0.736067	0.00119	-0.04373	0.049282	0.846	0.969304
ZP4	-0.01899	0.672477	0.79628	0.000698	-0.02964	0.034802	0.846	0.969304
POMC	-0.01814	0.840189	0.893885	-0.00266	-0.07882	0.043668	0.84	0.969304
METAP1	-0.00918	0.812096	0.883508	-0.00033	-0.02738	0.014905	0.858	0.971256
GAST	0.027839	0.845679	0.895702	-0.00545	-0.14435	0.081482	0.856	0.971256
ACADM	0.009611	0.915655	0.932123	-0.00063	-0.05262	0.023424	0.854	0.971256
PFDN4	-0.0534	0.468201	0.649514	-0.00057	-0.0487	0.023212	0.864	0.974151
TFPI2	0.184539	2.73E-05	0.000368	0.002927	-0.06063	0.069412	0.894	0.980628
MKI67	0.078541	0.115379	0.263325	0.001019	-0.04558	0.049184	0.886	0.980628
ITGAX	0.083211	0.232607	0.407634	-0.00126	-0.07262	0.063419	0.878	0.980628
MN1	-0.05649	0.452872	0.642437	0.000393	-0.03387	0.030317	0.888	0.980628
FASLG	0.021789	0.629641	0.764757	0.004258	-0.09122	0.093406	0.888	0.980628
SDHB	-0.03582	0.678126	0.797779	-0.00029	-0.03974	0.020094	0.894	0.980628
WARS	0.014353	0.775771	0.867759	0.003633	-0.08121	0.078241	0.878	0.980628
RNF4	0.081123	0.36517	0.566675	0.000353	-0.03972	0.034465	0.912	0.992677
IGFBP7	-0.01694	0.606146	0.752366	-0.00264	-0.11786	0.077518	0.912	0.992677
ZPR1	-0.11274	0.393307	0.592052	0.000187	-0.03013	0.025829	0.924	0.994266
MAP1LC3A	-0.02295	0.582614	0.73607	0.00029	-0.0268	0.029973	0.922	0.994266
LYZL2	-0.02137	0.646595	0.781993	0.000506	-0.04	0.046366	0.924	0.994266
HMCN2	0.098465	0.191722	0.369098	-0.00021	-0.03459	0.024427	0.93	0.996932
CRX	0.233976	0.075851	0.201301	0.000203	-0.02895	0.031825	0.938	1
COQ7	0.05386	0.351433	0.555618	5.38E-07	-0.02214	0.01727	1	1
AFP	0.072102	0.383119	0.582918	-3.22E-05	-0.01508	0.013136	0.97	1
ARNT	0.038716	0.409784	0.600874	-3.31E-05	-0.02014	0.014649	0.984	1
RNF43	0.053889	0.505242	0.677647	0.000738	-0.09776	0.089971	0.968	1
IL5RA	-0.03556	0.577268	0.736067	-4.71E-05	-0.01654	0.011174	0.962	1

Table S14 continued

Protein	β of UPF	*p*_value	q_value	proportion	LCI	UCI	*p*_value	q_value
GPR101	0.041843	0.602865	0.75159	9.72E-05	-0.02729	0.025908	0.952	1
PYDC1	0.028286	0.612257	0.756632	-8.48E-05	-0.02492	0.01663	0.968	1
LMOD2	-0.04298	0.618504	0.759674	1.27E-05	-0.03484	0.035151	0.992	1
SPINT1	-0.01295	0.655125	0.788938	-2.16E-06	-0.01873	0.014298	0.994	1
CDC25A	-0.02283	0.685019	0.797779	-0.00015	-0.04577	0.03905	0.954	1
ATP6AP2	0.014908	0.779443	0.868434	7.49E-07	-0.01786	0.011818	0.998	1
LSM8	-0.03104	0.798194	0.882379	-1.18E-05	-0.04608	0.027232	0.992	1
ADGRV1	-0.02001	0.82829	0.891277	0.000118	-0.02577	0.022604	0.956	1
JUN	0.013421	0.836408	0.89322	-2.34E-05	-0.01825	0.013653	0.986	1
KITLG	0.007891	0.85351	0.897169	0.001756	-0.19392	0.152094	0.962	1
ADGRB3	-0.0072	0.855956	0.897169	-8.64E-05	-0.03798	0.034038	0.99	1
STX5	0.009017	0.865619	0.903949	3.87E-05	-0.02981	0.020979	0.992	1
CEP112	0.004475	0.938789	0.942118	0.000107	-0.03246	0.029459	0.97	1

Abbreviations: LCI, lower confidence interval; UCI, upper confidence interval.

*: in mediation package, the *p* value less than 2.2*10^(-16) was shown as <0.0001.

#: after FDR adjustment, those *p* value shown as <0.0001 was shown as <0.0001.

Table S15. Relative ratio (95% CI) of MASLD risk associated with berry intake

Model 1 was adjusted for age when completing the most recent dietary recall, age when completing the

	**Tertile 1**	**Tertile 2**	**Tertile 3**	***P* for trend**	**Per 50 gram increment**
Berry intake, median (IQR)	0 (0, 0)	50.0 (37.9, 67.5)	135.0 (105.0, 180.0)		
No. of participants	10,043	10,180	9,163		
No. of cases	2,584	2,062	1,725		
Model 1	0 (reference)	0.83 (0.79, 0.88)	0.82 (0.77, 0.86)	<.0001	0.97 (0.95, 0.98)
Model 2	0 (reference)	0.90 (0.86, 0.95)	0.91 (0.86, 0.96)	0.0019	0.99 (0.98, 1.01)

MRI scan, and sex.

Model 2 was adjusted for variables in model 1 and race, education, socioeconomic status, smoking status, alcohol consumption, physical activity, total energy intake, and ultra-processed food intake quintiles.

Abbreviations: CI, confidence interval; IQR; interquartile range; MASLD, metabolic-dysfunction associated steatotic liver disease.

Table S16. β-coefficients (95%CI) of PDFF and liver cT1 associated with berry intake

	Tertile 1	Tertile 2	Tertile 3	*P* for trend	Per 50 gram increment
**Berry intake, median (IQR)**	0 (0, 0)	50.0 (37.9, 67.5)	135.0 (105.0, 180.0)		
**PDFF, median (IQR)**	2.97 (2.00, 5.21)	2.69 (1.87, 4.43)	2.56 (1.80, 4.15)		
No. of participants	10,043	10,180	9,163		
Model 1	0 (reference)	-0.41 (-0.52, -0.30)	-0.50 (-0.61, -0.38)	<.0001	-0.08 (-0.12, -0.05)
Model 2	0 (reference)	-0.24 (-0.35, -0.13)	-0.25 (-0.36, -0.13)	0.0002	-0.03 (-0.06, -0.0008)
**Liver cT1, median (IQR)**	696 (664, 733)	691 (661, 725)	689 (658, 723)		
No. of participants	6,344	6,566	5,864		
Model 1	0 (reference)	-4.73 (-6.59, -2.88)	-5.33 (-7.27, -3.39)	<.0001	-0.89 (-4.41, -0.38)
Model 2	0 (reference)	-2.90 (-4.74, -1.06)	-2.69 (-4.64, -0.74)	0.017	-0.35 (-0.87, 0.17)

Model 1 was adjusted for age when completing the most recent dietary recall, age when completing the MRI scan, and sex.

Model 2 was adjusted for variables in model 1 and race, education, socioeconomic status, smoking status, alcohol consumption, physical activity, total energy intake, and ultra-processed food intake quintiles.

Abbreviations: CI, confidence interval; IQR; interquartile range; PDFF, proton-density fat fraction.

Table S17. Stratified analyses by berry intake of association between UPF and PF intake and MASLD risk*

**Tertile 1**	**UPF**	**PF**
Quintile 1	1.00 (reference)	1.00 (reference)
Quintile 2	1.04 (0.93, 1.16)	0.98 (0.88, 1.09)
Quintile 3	1.21 (1.09, 1.35)	0.90 (0.80, 1.02)
Quintile 4	1.14 (1.02, 1.27)	0.96 (0.85, 1.10)
Quintile 5	1.38 (1.24, 1.54)	0.97 (0.84, 1.12)
P for trend	<.0001	0.87
Per 10% increment	1.07 (1.05, 1.10)	1.03 (0.998, 1.07)
**Tertile 2**		
Quintile 1	1.00 (reference)	1.00 (reference)
Quintile 2	1.10 (0.96, 1.25)	0.97 (0.85, 1.10)
Quintile 3	1.13 (0.99, 1.28)	0.90 (0.78, 1.03)
Quintile 4	1.25 (1.10, 1.42)	0.94 (0.80, 1.09)
Quintile 5	1.39 (1.23, 1.57)	1.01 (0.85, 1.20)
P for trend	<.0001	0.49
Per 10% increment	1.08 (1.05, 1.11)	1.05 (1.004, 1.10)
**Tertile 3**		
Quintile 1	1.00 (reference)	1.00 (reference)
Quintile 2	1.22 (1.05, 1.41)	0.99 (0.86, 1.12)
Quintile 3	1.26 (1.09, 1.46)	0.91 (0.79, 1.06)
Quintile 4	1.36 (1.18, 1.57)	1.05 (0.89, 1.23)
Quintile 5	1.57 (1.37, 1.81)	1.12 (0.93, 1.36)
P for trend	<.0001	0.13
Per 10% increment	1.11 (1.08, 1.14)	1.06 (0.99, 1.12)

***P* for interaction**	0.018	0.53

Data are shown in relative risk (95% CI)

Models were adjusted for age when completing the most recent dietary recall, age when completing the MRI scan, sex, race, education, socioeconomic status, smoking status, alcohol consumption, physical activity, and total energy intake.

Abbreviations: CI, confidence interval; MASLD, metabolic-dysfunction associated steatotic liver disease; MET, metabolic equivalent of task; PF, processed food; UPF, ultra-processed food.
